# Progress in NiO Based Materials for Electrochemical Sensing Applications

**DOI:** 10.3390/bios15100678

**Published:** 2025-10-09

**Authors:** Praveen Kumar, Mohammad Aslam, Saood Ali, Khaled Hamdy, Khursheed Ahmad, Danishuddin

**Affiliations:** 1 Department of Chemistry, Indian Institute of Technology Indore, Simrol 453552, MP, India; 2 School of Chemical Engineering, Yeungnam University, 280 Daehak-Ro, Gyeongsan 38541, Republic of Korea; 3 School of Mechanical Engineering, Yeungnam University, Gyeongsan 38541, Republic of Korea; 4 Department of Biotechnology, Yeungnam University, Gyeongsan 38541, Republic of Korea

**Keywords:** NiO, electrochemical sensors, biomolecules, toxic substances, environmental

## Abstract

Nickel oxide (NiO), a wide bandgap p-type semiconductor, has emerged as a promising material for electrochemical sensing owing to its excellent redox properties, chemical stability, and facile synthesis. Its strong electrocatalytic activity enables effective detection of diverse analytes, including glucose, hydrogen peroxide, environmental pollutants, and biomolecules. Advances in nanotechnology have enabled the development of NiO-based nanostructures such as nanoparticles, nanowires, and nanoflakes, which offer enhanced surface area and improved electron transfer. Integration with conductive materials like graphene, carbon nanotubes, and metal–organic frameworks (MOFs) further enhance sensor performance through synergistic effects. Innovations in synthesis techniques, including hydrothermal, sol–gel, and green approaches, have expanded the applicability of NiO in next-generation sensing platforms. This review summarizes recent progress in the structural engineering, composite formation, and electrochemical mechanisms of NiO-based materials for advanced electrochemical sensing applications.

## 1. Introduction

Electrochemical sensing technologies have attracted considerable attention in recent years due to their exceptional sensitivity, selectivity, low detection limits, portability, and rapid response time. These technologies play a crucial role in various fields, including clinical diagnostics, environmental monitoring, food quality control, and pharmaceutical analysis. The performance of electrochemical sensors is largely dependent on the properties of the sensing materials employed. Among a wide range of materials, transition metal oxides have emerged as promising candidates due to their rich redox chemistry, high thermal and chemical stability, and tunable electronic structures [1,2,3]. Among these, nickel oxide (NiO) has garnered particular interest for its favorable electrochemical characteristics. NiO is a p-type semiconductor with a wide bandgap of approximately 3.6–4.0 eV, and its intrinsic redox behavior, ease of fabrication, abundance, and environmental compatibility make it an ideal material for electrochemical applications [4,5,6]. Moreover, NiO exhibits excellent catalytic activity toward redox reactions, which is essential for sensitive and selective detection of various analytes such as glucose, hydrogen peroxide (H_2_O_2_), dopamine, urea, and toxic heavy metals [7,8,9]. These features render NiO highly attractive for constructing non-enzymatic electrochemical sensors, which avoid the limitations associated with enzyme-based sensors such as high cost, instability, and susceptibility to environmental factors.

Recent years have witnessed substantial progress in the synthesis and functionalization of NiO-based nanostructures for sensor applications. Nanostructured NiO, owing to its high surface area, tailored morphology, and enhanced active sites, provides increased electroactive surface area and improved mass transport, which are vital for enhancing sensor sensitivity. Various morphologies such as nanoparticles, nanowires, nanorods, nanoflakes, nanosheets, and hollow spheres have been synthesized using different physical and chemical approaches. Techniques such as hydrothermal synthesis, sol–gel processing, microwave-assisted synthesis, electrodeposition, and chemical vapor deposition (CVD) have enabled precise control over the structure and composition of NiO nanomaterials [10,11,12,13]. However, pristine NiO sometimes suffers from limitations such as low intrinsic electrical conductivity and sluggish electron transfer kinetics, which can hinder the overall sensing performance [14]. To overcome these limitations, NiO-based composites and hybrid materials have been extensively developed by combining NiO with other conductive and electrochemically active materials. These include carbon-based materials like graphene, reduced graphene oxide (rGO), carbon nanotubes (CNTs), conducting polymers (e.g., polyaniline, polypyrrole), noble metals (e.g., Au, Pt, Ag), and other metal oxides (e.g., ZnO, CuO, Fe_3_O_4_). These composite materials exhibit synergistic effects, improving electron transfer rate, enhancing electrochemical response, and providing better structural stability. Likewise, NiO/rGO nanocomposites show enhanced conductivity and catalytic performance due to the large surface area and high electron mobility of rGO. Similarly, NiO-MOF (metal–organic framework) hybrids combine the high porosity and tunable chemistry of MOFs with the electrochemical activity of NiO, making them excellent candidates for ultrasensitive sensor applications. Furthermore, NiO-decorated MXenes and NiO-cargraphitic carbon nitride (g-CN) composites have been explored to achieve higher electrocatalytic activity, long-term stability, and interference resistance [15,16,17,18]. Another significant advancement in the development of NiO-based electrochemical sensors is the tuning of surface defects, oxygen vacancies, and doping with heteroatoms or metals (e.g., Li^+^, Co^2+^, or rare earth elements). These modifications improve charge carrier density and modulate the electronic structure of NiO, thereby enhancing the sensing performance [19]. Doping strategies also help in tailoring the bandgap and redox behavior, which are crucial for analyte-specific interactions and detection in complex matrices [20]. The electrochemical mechanisms underlying the sensing process in NiO-based systems typically involve Faradaic redox reactions, where Ni^2+^/Ni^3+^ redox couples facilitate electron transfer during analyte oxidation or reduction [21]. In the case of glucose detection, for example, Ni^2+^ is oxidized to Ni^3+^ at the electrode surface, which subsequently oxidizes glucose molecules, generating an electrical signal proportional to the glucose concentration. This mechanism is particularly advantageous for non-enzymatic glucose sensing, offering high stability and reproducibility compared to enzymatic counterparts [22].

This review article discussed the progress in the fabrication of NiO based hybrid composites and their role as catalyst for the detection of various biomolecules and toxic substances and other hazardous compounds.

## 2. Synthesis Methods for NiO

### 2.1. Hydrothermal/Solvothermal Method

NiO and its composite materials are frequently synthesized via the hydrothermal/solvothermal method, which is favored due to its operational simplicity, adaptability, and ability to yield highly crystalline and uniformly distributed nanostructures at relatively low processing temperatures. In contrast, a green synthesis route was employed in one approach, wherein 7 g of nickel nitrate hexahydrate (Ni(NO_3_)_2_·6H_2_O) (NNH) was dissolved in 50 mL of deionized water. Subsequently, 20 mL of a natural plant-based surfactant specifically, extracts from orange blossom (S1), yarrow (S2), hibiscus tea (S3), green tea (S4), rosemary (S5), or barberry (S6) was introduced into the system. The pH of the resulting mixture was adjusted to neutral (pH 7) using 25% (*v*/*v*) ammonia solution, followed by continuous magnetic stirring for 48 h. The mixture then underwent ultrasonic treatment for 30 min before being sealed in a Teflon-lined autoclave and heated at 80 °C for 24 h. Upon natural cooling, the resulting solid was separated, washed with deionized water, and dried at 115 °C for 2 h. A subsequent calcination step at 410 °C for 10 h ensured the removal of residual organic content, producing gray-black NiO nanoparticles [23]. In another report, NiO was synthesized without the involvement of surfactants or template-directing agents, using a combination of NNH and urea (CH_4_N_2_O). A transparent solution was obtained by dissolving 0.16 M of NNH and 0.5 g of urea in 50 mL of deionized water, followed by stirring at 30 °C for 30 min to ensure homogeneity. This precursor solution was transferred into a Teflon-lined stainless-steel autoclave and subjected to hydrothermal treatment at 150 °C for 10 h. Post-reaction, the Teflon was allowed to cool naturally, and the resulting precipitate was separated by centrifugation, thoroughly washed with deionized water and ethanol, and air-dried at 50 °C for approximately 7 h. A final calcination step was performed at 500 °C for 2 h to obtain crystalline NiO [10]. Another method involved the hydrothermal synthesis of NiO from aqueous mixtures containing varied concentrations of nickel chloride and triethanolamine. Approximately 40 mL of these solutions were prepared and subjected to hydrothermal treatment in a Teflon-lined autoclave at 200 °C for 1 h. After natural cooling, the solid products were isolated through centrifugation, washed with deionized water, dried at 100 °C for 3 h, and calcined at 400 °C for 1 h to promote phase transformation into nickel(II) oxide [24]. In an alternative procedure, an aqueous solution of NNH was prepared by dissolving the precursor in 50 mL of distilled water under continuous stirring. Urea solution was added dropwise, followed by pH adjustment below 8 using ammonium hydroxide. The homogeneous mixture was stirred for an additional 30 min, transferred to a Teflon-lined stainless steel autoclave, and heated at 180 °C for 24 h. After cooling to room temperature (RT), the solid product was separated via centrifugation, washed multiple times with water and ethanol, dried overnight at 60 °C, and calcinated at 400 °C for 3 h to achieve phase-pure NiO nanoparticles [25]. Demir et al. employed polyvinylpyrrolidone (PVP) of varying molecular weights as stabilizers in the synthesis of NiO nanoparticles for antibacterial property investigations. A solution was prepared by dissolving 3 mmol of NNH and 0.200 g of PVP (10 kDa) in 100 mL of deionized water, followed by 15 min of vigorous stirring. Ammonium hydroxide was added dropwise to adjust the pH to 11, and the reaction mixture was stirred for an additional 30 min. The mixture was then sealed in a Teflon-lined autoclave and maintained at 120 °C for 12 h. The final product was recovered through centrifugation, washed thoroughly, dried at 60 °C overnight, and calcined at 400 °C for 2 h. In this study authors also optimized particle size by varying PVP molecular weights (K10, K15, K40), reaction times (4 to 12 h), and temperatures (60 to 140 °C) [26]. Qian et al. demonstrated a hydrothermal strategy to obtain flake-flower structured nickel oxide. They dissolved 0.475 g of nickel chloride hexahydrate in a binary solvent system consisting of 10 mL ethanol and 10 mL deionized water, followed by the addition of 0.2 g PVP and 10 mL ethylene glycol. The pH was adjusted to approximately 10 using aqueous ammonia, and after 5 min of stirring, the mixture was transferred to a Teflon-lined autoclave and heated for 10 h at 140 °C. The obtained greenish product was washed thoroughly and dried at 60 °C for 10 h, followed by calcination at 600 °C for 1 h to yield NiO [27]. Additionally, Cao and coworkers synthesized NiO nanobelts through a hydrothermal process. A precursor solution was prepared by dissolving 0.474 g of nickel chloride hexahydrate and 0.044 g sodium oxalate in 18 mL deionized water, followed by the addition of 30 mL ethylene glycol under vigorous stirring for 30 min. The homogeneous mixture was then transferred into a Teflon-lined autoclave and subjected to hydrothermal treatment at 180 °C for 12 h. After natural cooling, the product was recovered by centrifugation, repeatedly washed with ethanol and deionized water, dried at 60 °C for 24 h, and finally calcined at 400 °C for 2 h with a heating rate of 1 °C/min [28].

### 2.2. Microwave Method

Microwave-assisted synthesis has emerged as a highly effective strategy for fabricating NiO nanomaterials, offering significant advantages in terms of accelerated reaction rates, reduced energy consumption, and fine control over particle morphology. Utilizing microwave irradiation, which promotes rapid and uniform dielectric heating through direct interaction with polar or ionic species in the reaction solution, this technique fosters swift nucleation and crystal growth, thereby outperforming conventional heating methods. In one representative approach, nanoflake-structured NiO was synthesized without the need for any surfactants or additional additives. The procedure involved dissolving NNH in distilled water, vigorously stirring the resulting clear solution, and then incrementally introducing ammonia to achieve a pH of 9, yielding a transparent blue mixture. This solution was transferred into capped polypropylene autoclave bottles and exposed to microwave irradiation at varying power levels (300–900 W, 2.45 GHz) for 1 min. Cooling periods post-irradiation varied according to power setting, with higher-power samples requiring up to 45 min to return to ambient temperature. Precipitates were isolated by centrifugation, thoroughly washed with water and ethanol, then dried at 110 °C and subsequently calcined at 400 °C for 2 h. X-ray diffraction confirmed the formation of highly crystalline, cubic-phase NiO, with crystallinity increasing at higher microwave intensities. This additive-free, microwave-assisted route provides an efficient and reproducible pathway for preparing high-quality NiO nanostructures [29]. A variant of the microwave-assisted synthesis of NiO involves preparing a 24 mL aqueous solution of nickel acetate, which is magnetically stirred at room temperature for 15 min before preheating at 200 W to 60 °C. Upon reaching this temperature, 16 mL of 1 M NaOH is added dropwise. The resulting mixture is then heated under microwave irradiation at the chosen power setting until 90 °C is reached. Subsequently, an alternating cycle of 1 min microwave heating and 1 min rest is performed repeatedly to achieve a total synthesis duration of 10 min. After gradually cooling to room temperature overnight, the mixture is centrifuged and washed thoroughly with deionized water. The collected solid is dried at 90 °C for 18 h, finely ground, and divided for subsequent calcination at various temperatures and durations in atmospheric air [11]. Alternatively, solid-state microwave synthesis protocols employ analytical-grade nickel acetate or nickel formate as precursors, optionally blended with carbon black (in proportions of 5–15 wt%) to facilitate microwave absorption. The mixtures are thoroughly homogenized, pressed into 12 mm diameter pellets, and placed in alumina crucibles. For thermal uniformity and stabilization, mullite is used as an insulating support within the microwave chamber. The assembled crucibles are exposed to microwave irradiation (2450 MHz, 1000 W) for different durations ranging from 5 to 30 min, utilizing a standard domestic microwave oven. Following synthesis, NiO products are characterized for phase purity and structural features [30]. Recent advances demonstrate the versatility of microwave-assisted approaches for the synthesis of nickel oxide (NiO) nanostructures and their composites. For example, a simple one-pot strategy involved dissolving acrylamide and nickel sulfate hexahydrate in ethylene glycol, followed by ultrasonication and magnetic stirring to promote homogeneity. Rapid microwave irradiation at 480 W for 5 min enabled the in situ formation of polyacrylamide/NiO nanocomposites, where nickel salt decomposition and nanoparticle growth proceeded simultaneously. The resulting product was isolated by filtration, washed with ethanol, dried at 70 °C, and finely ground to a powder [31]. High-purity nickel oxide (NiO) nanoparticles with an average diameter of 15 ± 0.5 nm were synthesized via a solid-state microwave-assisted route using the [Ni(NH_3_)_6_](NO_3_)_2_ complex as a precursor. To obtain the complex, stoichiometric ammonia solution was added to an aqueous nickel nitrate hexahydrate solution under ice-cooling, followed by slow addition of cold ethanol and crystallization at low temperature for 2 h. The resulting crystals were isolated, washed with ethanol and ether, and dried under vacuum. For NiO formation, 2 g of the dried [Ni(NH_3_)_6_](NO_3_)_2_ was placed in a porcelain crucible nested within another crucible containing CuO powder as a microwave absorber. The assembly was irradiated in a 2.45 GHz, 900 W microwave oven in air, reaching up to 250 °C. Complete decomposition to NiO occurred within 10 min. The final product was cooled, washed with ethanol, and vacuum-dried over anhydrous CaCl_2_ [32]. Nickel oxide (NiO) nanoparticles with an average crystallite size of 15–16 nm were synthesized via a microwave-assisted, green combustion route utilizing Coriandrum sativum (coriander) leaf extract as a bio-fuel. In this method, stoichiometric amounts of NNH (0.581 g) were dissolved in water and mixed with the prepared coriander leaf extract in deionized water. After thorough stirring, the combined solution was placed in a silica crucible and irradiated in a domestic microwave oven (3.45 GHz, 950 W) for 10 min. The process induced rapid heating and spontaneous combustion, facilitating the decomposition of reactants and yielding solid NiO. The resulting powder was separated by centrifugation, washed multiple times with alcohol, dried at 120 °C for 3 h, and subsequently ground to a fine powder. This efficient, plant-assisted microwave technique enables rapid production of homogeneous, nanostructured NiO with minimal processing time and eco-friendly reagents [33]. Hierarchically porous NiO was produced via a microwave-assisted synthesis, where a solution of nickel nitrate, CTAB, and urea (Ni:urea = 1:2) was stirred thoroughly before being microwave-irradiated at 250 W and 120 °C for 15 min. The resulting precipitate was cooled, centrifuged, washed sequentially with water and ethanol, then vacuum-dried at 60 °C overnight. Final calcination at 300 °C for 3 h yielded NiO with ball-shaped, hierarchical porosity and ripple-like surface features [34].

### 2.3. Sol–Gel Method

Sol–gel processing represents a versatile chemical route for the synthesis of NiO nanomaterials, providing precise control over morphology, composition, and phase purity. A common approach involves dissolving nickel precursors, such as nickel acetate tetrahydrate or nickel nitrate hexahydrate, in polar solvents like ethanol or deionized water. To facilitate homogeneous dispersion, the solution is typically subjected to magnetic stirring, followed by pH adjustment using alkaline agents such as ammonium hydroxide (NH_4_OH), ensuring favorable conditions for hydrolysis and gelation processes. Various sol–gel techniques have been extensively employed for the synthesis of nickel oxide (NiO) materials, offering control over particle morphology, crystallinity, and size distribution. A common route involves the dissolution of nickel precursors, such as nickel acetate tetrahydrate, in alcoholic media (e.g., ethanol), followed by controlled stirring and pH adjustment. Typically, homogeneous mixing is ensured by magnetic stirring (e.g., 30 min) prior to refluxing at elevated temperatures (around 80 °C) for several hours. Gelation is often facilitated using polymeric additives like polyethylene glycol (PEG) and ammonium hydroxide to adjust the pH, generally maintained between 7.5 and 11. The resultant gel is aged, washed thoroughly, and calcined at temperatures ranging from 400 °C to 800 °C to eliminate organic residues and crystallize the oxide phase [12]. Biopolymer-assisted routes have also been explored, where gelatine serves as a stabilizing matrix. Nickel nitrate solutions are integrated into a gelatine matrix and aged at elevated temperatures (80 °C) to form a stable gel, which after pyrolysis at controlled temperatures (350–700 °C), yields phase-pure NiO nanoparticles [35]. In another approach, nickel nitrate hexahydrate and citric acid are dissolved in an alcohol medium, followed by evaporation at ~120 °C to yield a xerogel or paste. This intermediate is subjected to high-temperature calcination (up to 750 °C) to generate NiO nanowires, with citric acid acting as a chelating agent that promotes the formation of single-crystalline hexagonal nanostructures via vapor–solid (VS) mechanisms [36]. Alternative methods employ surfactants, such as Triton X-100 or Tween 20, as structure-directing agents to prevent particle agglomeration and regulate nanoparticle formation. For example, nickel sulfate heptahydrate is reacted with Triton X-100 and ammonium hydroxide, maintaining the pH around 8, to obtain a sol that transitions to gel upon aging. Subsequent calcination at 400 °C yields fine NiO powders [37]. Similarly, NNH with additives like acetic acid, Tween 20 and hydrochloric acid in ethanol forms homogeneous after 2 h of stirring, upon calcination at 500 °C, produce supported NiO composites, including binary systems such as NiO-TiO_2_ or NiO-CeO_2_ [38]. Other formulations include co-solvent systems such as isopropanol and PEG, with the pH adjusted to basic conditions (pH 11) via ammonium hydroxide, followed by low-temperature drying and calcination [39]. Methanol/water mixtures have also been utilized where nickel acetate undergoes sol–gel conversion under mild heating (60 °C) with final calcination at 350 °C, resulting in porous NiO nanosheets [40]. Citric acid and ethylene glycol systems are another popular choice, where nickel acetate is combined with citric acid and polyol, promoting polyesterification reactions and gelation at ~90 °C. A two-step calcination process (pre-calcination at 250 °C, final at 800 °C) is often applied to achieve high-purity NiO with reduced organic contamination [41]. Stabilizer-assisted sol–gel approaches, using ethanol and ligands like monoethanolamine (MEA) or ethylenediamine (en), are employed to control hydrolysis kinetics and prevent premature gelation, effectively producing compact or porous NiO films [42]. Additionally, pH-controlled precipitation from aqueous nickel acetate solutions followed by gel formation, drying, and calcination (or even microwave-assisted heating) allows for tuning of particle morphology and phase formation, with the final product properties being highly dependent on the synthesis pH and calcination conditions [43].

### 2.4. Co-Precipitation Method

Nickel oxide (NiO) nanoparticles have been widely synthesized via co-precipitation techniques employing various nickel salts and alkaline precipitating agents. This method remains a preferred route due to its simplicity, cost-effectiveness, and ability to produce high-purity nanoparticles with controlled morphology. Collectively, there were various co-precipitation methodologies highlight the versatility of this synthesis route in tailoring the structural, morphological, and crystallographic properties of NiO nanoparticles by modulating process parameters such as pH, temperature, calcination protocol, and the use of capping or surfactant agents. In one approach, NiO nanoparticles were synthesized using NNH as the Ni^2+^ precursor and ammonium hydroxide (NH_4_OH) as the hydroxide ion source. The synthesis was carried out by dissolving a stoichiometric amount of NNH in deionized water and stirring the solution at ~45 °C with a constant stirring speed of 300 rpm. The pH was maintained at approximately 4.0 by the controlled addition of 0.2 M NH_4_OH over a period of 2 h. Upon the appearance of light green nickel hydroxide [Ni(OH)_2_] precipitates, the addition of NH_4_OH was stopped. The precipitate was filtered, washed repeatedly with deionized water and ethanol, and dried at 100 °C for 4 h. Subsequent calcination at 500 °C for 4 h resulted in around 2.5 g of phase-pure NiO nanoparticles. The gradual thermal treatment from RT to 500 °C at a ramp rate of 5 °C/min allowed for improved crystallinity and minimized agglomeration, demonstrating decent structural properties compared to conventional methods [44]. Another method involved using nickel acetate tetrahydrate (Ni(CH_3_COO)_2_·4H_2_O) and sodium hydroxide (NaOH) with a fixed molar ratio of 1:4. After dissolving both reagents in distilled water separately, the NaOH solution was gradually introduced into the nickel solution under constant magnetic stirring and mild heating. The resultant nickel hydroxide precipitate was collected, washed to remove residual ions, dried at 90 °C, and subjected to high-temperature calcination at 800 °C for 2 h to obtain well-crystallized NiO nanoparticles [45]. In another study, NiO was synthesized via a co-precipitation method, wherein separate aqueous solutions of NNH and NaOH were prepared and subsequently mixed dropwise under continuous stirring at approximately 50 °C. The precipitate was washed, filtered, and dried at 150 °C for 11 h, followed by calcination at 300 °C for 2 h. The final NiO powder, after thorough grinding, was characterized for morphological and structural properties [46]. Further, a modified co-precipitation method incorporated ethylenediaminetetraacetic acid (EDTA) as a capping agent to control particle agglomeration. Nickel chloride hexahydrate (NiCl_2_·6H_2_O) and EDTA were dissolved to obtain 1 M and 0.1 M solutions, respectively. NaOH (4 M) was titrated into this solution until a pH of 13 was achieved, and precipitation was conducted at different temperatures (60, 70, and 80 °C) to investigate the thermal effects on particle formation. The resulting precipitates were washed to neutral pH, dried, and calcined at 550 °C for 4 h, producing phase-pure NiO nanoparticles. The synthesized samples exhibited distinct characteristics depending on the reaction temperature, providing insight into thermal effects on NiO formation [47]. An alternative method employed sodium peroxide (Na_2_O_2_) as an oxidizing and precipitating agent. Equimolar aqueous solutions of nickel nitrate and Na_2_O_2_ were mixed rapidly at RT without stirring, followed by static aging overnight. The resulting apple-green precipitate was isolated by centrifugation, thoroughly washed, vacuum-dried at 60 °C, and calcined at 250 °C to yield fine NiO nanoparticles with distinct nano-structural features [48]. Another technique utilized a cationic surfactant, cetyltrimethylammonium bromide (CTAB) as a surfactant to influence nanoparticle morphology. Nickel chloride was dissolved in distilled water, combined with CTAB solution, and stirred prior to the dropwise addition of NaOH. The appearance of a light green precipitate marked the formation of nickel hydroxide, which was then isolated, washed with ethanol, dried at ~50 °C, and calcined at ~400 °C to yield black NiO nanopowder with potentially tailored surface properties [49].

### 2.5. Sputtering Method

Nickel oxide (NiO) thin films have been extensively fabricated through advanced sputtering techniques owing to their superior structural, optical, and electrochemical functionalities. Among these, direct current (DC), radio frequency (RF), and reactive magnetron sputtering approaches have been primarily employed, facilitating precise control over film morphology, crystallinity, and stoichiometry. In one common approach, NiO_x_ thin films were deposited onto 2 mm thick glass substrates, both bare and coated with indium-doped tin oxide (ITO), utilizing reactive DC magnetron sputtering. A high-purity circular nickel target (diameter 76.5 mm) was employed in a multi-target sputtering chamber. Argon (Ar) gas at a flow rate of 45 standard cubic centimeters per min (sccm) served as the sputtering medium, while high-purity oxygen (O_2_) gas was introduced as the reactive component with flow rates ranging from 0 to 15 sccm, under a maintained total working pressure of ~4 Pa. Sputtering was performed at a constant discharge power of 200 W. Process parameters such as discharge voltage and current were monitored with respect to oxygen flow variations. The resultant ~200 nm NiO_x_ layer was further integrated into multi-stack electrochromic devices (ECDs), comprising sequential depositions of Ta_2_O_5_ solid electrolytes (~350 nm) and WO_3_ electrochromic layers (~300 nm), finalized by a secondary ITO electrode, all fabricated via reactive sputtering [50]. NiO_x_ thin films have also been fabricated on p^+^-n silicon substrates and electrochemical quartz crystal microbalance (EQCM) sensors through reactive sputtering. Prior to deposition, EQCM substrates underwent ultrasonic treatment in acetone, isopropanol, and ultrapure water (18.2 MΩ cm). The sputtering process was executed at 573 K, 3 mTorr pressure, 140 W power, and an Ar:O_2_ flow ratio of 5:2, using a high-purity metallic Ni target. The gold-coated EQCM crystals ensured electrochemical activity, while on silicon wafers, a pre-sputtered thin Ni layer (~5 nm) prevented surface oxidation during NiO_x_ growth [51]. Nanostructured NiO thin films were prepared via RF magnetron sputtering under varying process conditions, including deposition pressures (20–40 mTorr), substrate temperatures (ambient to 200 °C), and oxygen partial pressures (60–100% O_2_ in Ar-O_2_ mixtures). High-purity (99.9999%) nickel foil targets were employed, and films were deposited on ITO-coated glass substrates, pre-cleaned through sequential ultrasonication in trichloroethylene, acetone, and isopropanol. A consistent target-to-substrate distance of 6 cm and RF power of 40 W were maintained. Gold (Au) top contacts were fabricated by thermal evaporation for device applications [52]. Custom-built DC magnetron sputtering systems have been utilized for NiO thin film deposition on Corning 7059 glass substrates. The systems achieved ultimate vacuum pressures of 5 × 10^−4^ Pa, using a combination of rotary and diffusion pumps. Sputtering was performed from high-purity Ni targets (99.98%, diameter 100 mm), with individual flow regulation of Ar and O_2_ gases via mass flow controllers. Sputtering power was varied (100–200 W), while other parameters such as total pressure and substrate temperature remained fixed [53]. Reactive RF sputtering has also been employed for the growth of Ni/NiO bilayer films on stainless steel (304 SS) substrates. Substrates were cleaned through a combination of detergent washing and ultrasonic treatment in organic solvents. Depositions were performed at 70 W sputtering power, 250 °C substrate temperature, 5 × 10^−3^ Torr chamber pressure, and varying O_2_ flows (0–20 sccm), with Ar flow fixed at 80 sccm, producing electrodes with tunable oxygen incorporation [54]. In studies focusing on electrochromic applications, ITO glass (20 × 20 mm^2^, ~84% transmittance) was used as the substrate, while Ni (99.995%) and ZnO (99.99%) targets were employed in DC magnetron sputtering systems (JGP-450). Substrate-target separation was 11 cm, and the base pressure was maintained at 9.8 × 10^−4^ Pa. Oxygen and argon gas flows were regulated via flow controllers, and optimized sputtering power (150 W) was found to yield superior electrochromic behavior [55]. For modified functionalization, Cu-doped NiO films were synthesized using co-sputtering techniques. NiO and Cu targets (99.99% purity) were co-deposited on ITO substrates under RF sputtering (40 W for NiO and 15 W for Cu), with controlled Cu incorporation achieved by varying deposition durations. Additionally, porous NiO films were fabricated on conductive Ni foam substrates and subsequently treated through rapid thermal annealing (RTA) at 400 °C to enhance structural and electrochemical performance. [56] Further optimization involved NiO films deposited via reactive DC magnetron sputtering on ITO glass (20 × 20 mm^2^). The substrates were ultrasonically cleaned, and the deposition was conducted at a target-to-substrate distance of 12 cm under a working pressure of 1.0 × 10^−3^ Pa. A pre-sputtering step (5–10 min) was employed to clean the target surface prior to film growth [57]. A typical RF magnetron sputtering process for NiO film deposition involves the use of a high-purity NiO target (≥99.995%) onto a glass substrate. The process parameters are finely tuned to control film composition and morphology. In one such study, a 100 nm thick NiO film was deposited using a sputtering gas mixture of argon and oxygen, with the oxygen partial pressure modulated over a full range (0–100%) to investigate its influence on the film properties. Deposition was conducted at RT (27 °C), using a power of 100 W and a pressure of 4 × 10^−2^ Torr, while maintaining a substrate-target distance of 5.5 cm and a growth rate of 10 nm/min. For device integration, such films have been employed in the fabrication of inverted-staggered thin-film transistors (TFTs). A 300 nm thick aluminum gate electrode was sputtered and patterned, followed by a 200 nm thick silicon oxide (SiO_2_) gate dielectric layer deposited via plasma-enhanced chemical vapor deposition (PECVD), utilizing silane and nitrous oxide precursors. A 50 nm NiO active layer was deposited using a lift-off technique, and source/drain contacts (300 nm Al) were similarly defined. Final annealing was carried out at 200 °C in ambient conditions for 30 min to improve film quality and interface characteristics [58].

### 2.6. Physical Vapor Deposition Method

The development of high-quality nickel oxide (NiO) thin films has gained considerable attention for applications in electronics and optoelectronic devices. Porous NiO thin films were fabricated via pulsed laser deposition (PLD) in a stainless steel vacuum chamber evacuated to ~1 × 10^−4^ Pa. A Q-switched Nd:YAG laser (1064 nm, 10 Hz, 8 ns pulse width) was focused onto a rotating Ni target, generating a plasma plume that reacted with O_2_ gas introduced at 50 Pa. Deposition was performed at RT with a target-to-substrate distance of 3 cm. Films were deposited on Au-coated Ti substrates for electrochemical studies and on Si substrates for structural characterization. Film mass was determined gravimetrically using a 0.01 mg-precision electrobalance [59]. Whereas, Pulsed Nd:YAG laser irradiation (1064 nm, 10 Hz repetition rate, 18 ns pulse width) with power levels between 410 and 820 mW was employed in an ultrahigh-vacuum (UHV) PLD system consisting of a stainless steel chamber (15 cm diameter, 25 cm height). The laser beam was focused onto a rotating (36 rpm) high-purity (3N) Ni target (1 inch diameter, 0.125 inch thickness) at a 45° incidence angle using a 75 cm focal length lens. The chamber was evacuated to a base pressure of ~3–5 × 10^−8^ Torr via turbo-molecular and ion pumps. O_2_ (99.99% purity) was introduced through an ultra-precision leak valve to maintain partial pressures between 0.01 and 1 Torr during deposition. The target-to-substrate distance was maintained at approximately 4 cm. NiO films were deposited on silicon (Si(100), n-type, 500 μm thick, 5 × 15 mm^2^) and glass substrates, with the latter cleaned ultrasonically in acetone, methanol, and deionized water for 5 min each before deposition. Substrates were loaded into the chamber after reaching the minimum achievable pressure [60].

In one approach, nanocrystalline NiO thin films were deposited using a vacuum-assisted e-beam evaporation technique. The high-purity NiO powder (99.99%) was compressed into 12 mm pellets and sintered at 1300 °C. Cleaned glass substrates (75 mm × 25 mm × 1.35 mm) were mounted in a vacuum chamber evacuated to 4 × 10^−6^ mbar. The NiO pellets, placed in a graphite crucible on a water-cooled copper hearth, were evaporated using a 5 kV electron beam and 50 mA filament current. Deposition was carried out at a chamber pressure of 5 × 10^−4^ mbar for 5 min, with a growth rate maintained at 1 nm/s. Thickness monitoring was performed using a quartz crystal microbalance [61]. In another study focused on optimizing deposition conditions, NiO films of 100 nm thickness were deposited onto 4-inch soda lime glass substrates using an e-beam evaporator operating at 1 Å/s. Substrates were ultrasonically cleaned in isopropanol and deionized water, then dried with nitrogen gas. The chamber was evacuated to a base pressure of 3.5 × 10^−6^ Torr. Deposition was conducted at two working pressures (2 × 10^−4^ Torr and 2 × 10^−5^ Torr) in a reactive oxygen atmosphere, with substrates rotating at 5 rpm and positioned 100 mm from the source to ensure film uniformity [62]. Electron beam evaporation was also employed to deposit NiO films onto ITO-coated glass substrates. High-purity NiO powder (<325 mesh, 99.99%) was pressed into 11 mm diameter, 4 mm thick pellets and sintered at 1300 °C. The films were deposited at a base vacuum of 7 × 10^−7^ Torr and a working pressure of 1 × 10^−5^ Torr. Substrates were placed parallel to the target at a 130 mm distance. Films with different thicknesses (170–540 nm) were fabricated by varying the deposition time accordingly [63]. Further variations in NiO film deposition using e-beam evaporation were investigated under different power settings and deposition times. NiO targets were prepared by pressing the powder into pellets using a specially designed die and sintered at 1300 °C. The e-beam chamber was evacuated to 9.3 × 10^−5^ Pa before deposition. The working voltage was held constant at 4 kV while varying the power and duration to evaluate film characteristics. These films were subsequently analyzed to assess their optical, structural, and electronic performance [64]. Doping of NiO with zinc (Zn) enhances its electronic properties and sensor performance. Zn-doped NiO thin films have been fabricated using an e-beam evaporation system by first preparing 10 wt% Zn:NiO targets through a solid-state synthesis route. High-purity NiO and ZnO powders were homogenized in ethanol and calcined at 500 °C for 4 h to ensure phase purity and prevent shrinkage during sintering. The calcined powders were pressed into pellets (12 mm diameter, 8 mm thickness) using uniaxial hydraulic pressing (5 tons/cm^2^) and sintered at 1300 °C for 5 h. These pellets served as targets in the e-beam deposition system for the preparation of Zn:NiO films [65].

### 2.7. Chemical Vapor Deposition Method

Chemical vapor deposition (CVD) is a widely employed method for fabricating high-quality NiO thin films with controlled thickness and crystallinity. In a typical approach, a custom-designed horizontal cold-wall CVD reactor equipped with a quartz reaction chamber and resistively heated metal susceptor is utilized. Depositions are carried out on pre-cleaned silicon (Si(100)) substrates with dimensions of 1 × 1 cm^2^. Nickel precursors, such as Ni(tfa)_2_·TMEDA, Ni(fod)_2_·TMEDA, and Ni(thd)_2_·TMEDA, are volatilized at optimized temperatures (75 °C, 80 °C, and 120 °C, respectively) in external glass vessels. Precursor vapors are transported into the reactor using high-purity oxygen gas, while an auxiliary oxygen flow passes through a water bath maintained at 35 °C to introduce moisture into the chamber. Both oxygen streams are regulated at a flow rate of 100 sccm using mass flow controllers. The depositions are performed at a constant substrate temperature of 400 °C under a total pressure of 10 mbar [66]. In another method, a cold-wall flow-type reactor is employed for NiO thin film growth using [Ni(dmamp′)_2_] as the precursor. The system is fully automated with precise control over gas flow rates, heating profiles, and pressure conditions. High-purity argon (99.998%) serves as the carrier gas, with flow rates ranging from 20 to 700 sccm. Substrates such as quartz slides are cut into pieces (approximately 4.0 × 2.5 cm), cleaned with isopropanol, and dried before use. Prior to deposition, the system is evacuated to a base pressure of ~4 × 10^−2^ mbar. The precursor is vaporized using a bubbler setup, with the outlet line heated to prevent condensation, and deposited under inert gas flow at pressures between 1 and 7 mbar until the desired film thickness is achieved [13]. A distinct CVD route involves using mesoporous Al_2_O_3_ gel as a substrate and bis(cyclopentadienyl)nickel (Ni(Cp)_2_) as the nickel source, with water vapor and oxygen serving as co-reactants. The solid precursor (2.5 g) is placed at the base of a stainless-steel reaction chamber, while the mesoporous substrate is suspended above it. The chamber is sealed and heated sequentially, first to 100 °C for 2 h to saturate with Ni vapor, followed by 260 °C for 13 h to facilitate NiO deposition [67]. Ultrasonic spray-assisted CVD has also been applied for NiO film preparation on fluorine-doped tin oxide (FTO) substrates. Nickel(II) acetylacetonate is dissolved in toluene with N,N-dimethylaminoethanol to enhance solubility and volatility. This precursor solution is atomized into fine aerosols via an ultrasonic humidifier. The aerosol is carried by an air stream through a multi-chamber system to deposit NiO films at 450 °C. Deposition time variations (10, 15, and 20 min) allow control over the film thickness, referred to as NiO(10 min), NiO(15 min), and NiO(20 min), respectively [68]. Additionally, plasma-assisted metal–organic CVD (PA-MOCVD) has been utilized to fabricate NiO films on sapphire substrates. Nickel MCP_2_ serves as the precursor, with high-purity argon and oxygen acting as carrier and reactive gases, respectively. Initial buffer layers are grown at 450 °C followed by deposition at elevated temperatures (510 °C, 530 °C, 570 °C, and 600 °C) under a constant pressure of 80 Pa. The films typically achieve a thickness around 200 nm, confirmed through profilometry measurements [69].

### 2.8. Laser Ablation Method

Pulsed Laser Ablation in Liquid (PLAL) is a promising, surfactant-free method for synthesizing nanostructured nickel oxide (NiO) particles, where the particle size, morphology, and optical properties can be effectively tuned by altering the laser parameters. In a typical PLAL process, nickel oxide nanoparticles were synthesized by ablating a pre-cleaned nickel target immersed in 5 mL of ultrapure water using a Q-switched Nd:YAG laser (λ = 1064 nm, pulse duration = 7 ns, repetition rate = 10 Hz), with laser fluences ranging between 50 and 150 mJ per pulse. The laser beam was focused using a plano-convex lens (70 mm focal length), and the ablation was performed under continuous mechanical stirring for 10 min [70]. In another approach, a nickel foil (99.95% purity, 2 mm thick) was immersed in 3% hydrogen peroxide solution and subjected to laser ablation using the third harmonic (355 nm) of an Nd:YAG laser operating at 10 Hz. The target was rotated during ablation to prevent deep cratering, with the laser focused via a 250 mm lens for 30 min. The resulting colloidal NiO nanoparticles were isolated through centrifugation [71]. NiO nanoparticles have also been synthesized using an Nd:YAG laser (1064 nm, 6 ns pulse duration, 10 Hz) with pulse energies between 400 and 800 mJ. A nickel plate was submerged in 3 mL of aqueous medium, and the laser beam was focused through a 10 cm lens for 5 min at RT (~27 °C), yielding nanoparticles with controlled size distributions [72]. Further studies employed pulsed laser irradiation of a nickel plate (99.9% purity) submerged in 50 mL of distilled water. After ultrasonic cleaning of the equipment, the target was irradiated using 1000 pulses of a Q-switched Nd:YAG laser (1064 nm, 7 ns, 10 Hz). A focal lens of 80 mm was used, producing a focused spot of approximately 15–20 µm on the target. Various fluences (0.6–1.2 J/cm^2^) were applied to control the size and properties of the NiO nanoparticles [73]. Another protocol involved ablating a nickel pellet (1.5 cm diameter, 3 mm thickness) submerged in 1 mL of deionized water, using a Q-switched Nd:YAG laser (1064 nm, 9 ns pulse width, 1 Hz repetition rate), with pulse energies between 40 and 200 mJ and varying ablation times from 5 to 20 min. The colloidal nanoparticle concentration was estimated by measuring the mass loss of the target [74]. High-power continuous wave fiber lasers have also been used for ablation of nickel plates (99.99% purity, 0.5 mm thick) in either deionized water or aqueous SDS solutions (0.001–0.1 M). A YLR-1000-SM fiber laser (1070 nm, ~250 W) was focused to a 40 µm spot size on the target, with 1 s irradiation cycles repeated multiple times. The colloidal suspensions were purified by high-speed centrifugation and washing to remove surfactant residues [75]. Another study used a top-down irradiation setup with a Q-switched Nd:YAG laser (1064 nm), where the laser power (5.8–10.5 W), repetition rate (0.1–8 kHz), and pulse duration (70–200 ns) were systematically varied. The beam, focused to ~100 µm diameter, ablated a cylindrical nickel pellet (99.995% purity) submerged in 5 mL deionized water, resulting in nanoparticle formation within 5 min [76]. Additionally, NiO-PVA nanocomposites were prepared by ablating a nickel target submerged in a 10 wt% polyvinyl alcohol (PVA) aqueous solution. A Q-switched Nd:YAG laser (1064 nm, 7 ns, 10 Hz, 150 mJ/pulse) was employed, focusing the beam to ~17.5 µm spot size using a 70 mm focal length lens and knife-edge technique. Ablation durations of 15, 30, and 60 min were used to control the composite properties, resulting in uniform NiO nanoparticle distribution within the PVA matrix [77].

## 3. Progress in Electrochemical Sensors

### 3.1. NiO/Graphene

**Figure 1:**
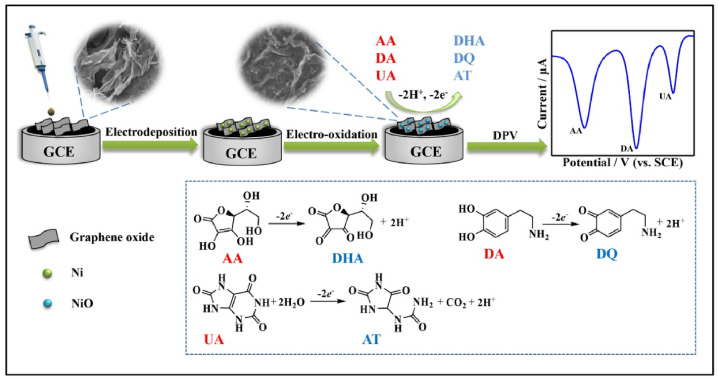
Schematic representation of the fabrication of GCE for electrochemical sensing of AA, DA and UA monitoring. Reprinted with permission [84].

Nickel oxide (NiO)-based nanocomposites integrated with graphene derivatives have garnered significant interest in recent years due to their exceptional electrochemical performance in the detection of biomolecules. Utilizing a hydrothermal method, Joseph et al. [78] synthesized metal oxide/reduced graphene oxide (MO/rGO) nanocomposites where MO comprised NiO, Co_3_O_4_, and SnO_2_. These composites were employed in fabricating modified carbon paste electrodes for the electrochemical detection of epinephrine (EPN), serotonin (SER), and tyrosine (TYR). The electrochemical behavior of these sensors was investigated using cyclic voltammetry (CV), differential pulse voltammetry (DPV), chronoamperometry, and electrochemical impedance spectroscopy (EIS). The enhanced electrocatalytic performance was attributed to the synergistic interaction between the high surface area and conductivity of rGO and the redox activity of the metal oxides. Among the composites, NiO/rGO exhibited superior sensitivity, with detection limits of 158 pM for EPN, 165 pM for SER, and 519 pM for TYR. In another study, a NiO/graphene nanosheet (NiO/GNS) composite was developed via a simple hydrothermal approach followed by thermal treatment. This material functioned as a highly active non-enzymatic electrocatalyst for glucose oxidation. The sensor demonstrated a broad linear response range (5 µM to 4.2 mM) and a low limit of detection (5.0 µM), outperforming bare NiO due to the improved electron transport and suppression of nanoparticle aggregation afforded by graphene integration. Furthermore, the composite was employed in glucose-powered biofuel cells, where it showed high current density and operational stability [79]. A novel non-enzymatic cholesterol sensor was fabricated by depositing Ni(OH)_2_ onto chemical vapor deposition (CVD)-grown graphene followed by thermal conversion to NiO. Morphological characterization confirmed the presence of flower-like NiO structures on the graphene surface. The sensor demonstrated remarkable performance, including high sensitivity (40.6 mA μM^−1^ cm^−2^), a detection limit of 0.13 μM, and a rapid response time of 5 s. The device effectively quantified cholesterol in milk samples and exhibited excellent resistance to interference from other biomolecules [15]. For detecting pethidine (PTD), a synthetic opioid, a NiO–GO nanocomposite-modified glassy carbon electrode (NiO-GO/GCE) was fabricated using a chemical reduction strategy. The developed sensor showed high sensitivity with a detection limit of 3.8 ng/mL and a wide linear range (0–500 μg/mL). Validation studies demonstrated its efficacy in human urine samples, with recoveries ranging from 90.00% to 99.66% and relative standard deviations below 5.41%, affirming its suitability for clinical monitoring [80]. To enable sensitive and rapid detection of dopamine (DA), a nanocomposite of oxygen-deficient NiO_x_ and partially reduced graphene oxide (p-rGO) was electrochemically deposited onto fluorine-doped tin oxide (FTO) substrates. The resulting electrode displayed excellent electrochemical properties, achieving a sensitivity of 24.76 µA µM^−1^ cm^−2^, a low detection limit of 22.0 nM, and a fast response time of 30 ms. This robust sensing platform demonstrated high selectivity, stability, and good recovery (98–100%) for dopamine detection in human urine samples [8]. An environmentally friendly approach was adopted to synthesize NiO nanoparticles using aqueous and ethanolic leaf extracts of Elettaria cardamomum, both with and without graphene oxide (GO). The resulting NiO-GO nanocomposites were evaluated for their electrochemical and photocatalytic properties. Among the synthesized materials, NiOet@GO exhibited the highest electrochemical capacitance and was most efficient in the photodegradation of crystal violet dye under sunlight, with degradation efficiency exceeding 90% [81]. A 2D porous NiO-rGO nanocomposite was synthesized via hydrothermal processing for epinephrine (EPI) detection. SEM and TEM analyses confirmed uniform dispersion of NiO nanoparticles on graphene sheets. The modified electrode (NiO-rGO/GCE) exhibited excellent electrocatalytic activity, attributed to enhanced charge transfer and surface conductivity. The sensor showed a linear response for EPI concentrations ranging from 50 μM to 1000 μM, with a correlation coefficient of 0.9986 and a detection limit of 10 μM. High recovery rates in biological fluids confirmed its practical applicability [82]. Additionally, a CuO–NiO/rGO nanocomposite synthesized via a sol–gel method was used for dopamine sensing. The composite modified electrode exhibited a diffusion coefficient of 1.04 × 10^−6^ cm^2^ s^−1^ and demonstrated excellent sensitivity (7.2 µA cm^−2^ mM^−1^) with a detection limit of 0.006 µM. Its application in wearable sensing devices was proposed for neurological monitoring, particularly in disorders such as Parkinson’s disease [83]. A multifunctional sensing platform for the simultaneous determination of uric acid (UA), dopamine (DA), and ascorbic acid (AA) was developed by electrodepositing NiO nanoparticles onto a graphene oxide-modified surface. The fabrication of the electrode has been described in Figure 1. The platform enabled distinct and well-separated redox peaks for each analyte. The sensor showed linear responses over wide concentration ranges with detection limits of 0.14 µM (UA), 0.10 µM (DA), and 5.50 µM (AA). High selectivity, reproducibility, and long-term stability were demonstrated in real sample analyses [84].

### 3.2. NiO/CNTs

**Figure 2:**
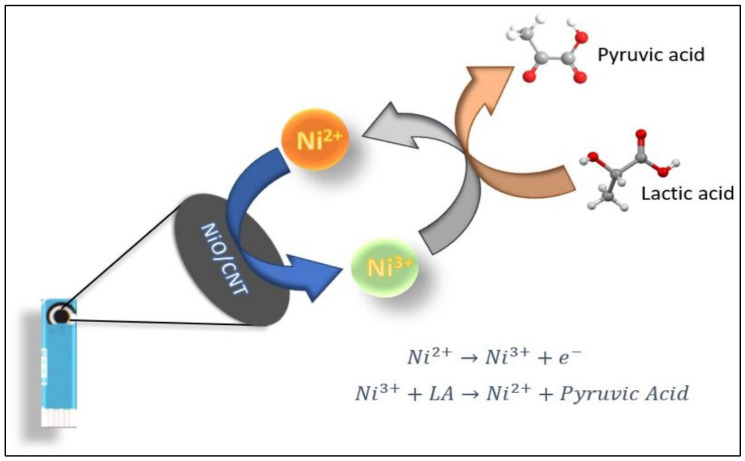
Schematic representation of the sensing of LA and its mechanism at fabricated electrode surface. Reprinted with permission [86].

Nickel oxide (NiO) nanoparticles integrated with carbon nanotubes (CNTs), especially multi-walled carbon nanotubes (MWCNTs), have emerged as promising materials for the development of high-performance electrochemical sensors. These hybrid systems leverage the redox-active nature of NiO and the excellent electrical conductivity and high surface area of CNTs, offering a synergistic platform for enhanced biosensing. A solvent-free approach has been employed to synthesize NiO-decorated MWCNT composites. The composite was used to fabricate an enzyme-free carbon paste electrode (CPE) for glucose detection. Among various formulations, the 10% NiO–MWCNT-based electrode demonstrated the best performance, achieving high sensitivities of 1696 and 122.1 µA·mM^−1^·cm^−2^ for glucose concentration ranges of 1–200 µM and 0.5–9.0 mM, respectively. The sensor also exhibited low detection limits (11.04 nM and 31 µM), along with high selectivity, reproducibility, and long-term stability, making it suitable for reliable non-enzymatic glucose monitoring [9]. A coaxial nanostructured composite of NiO, CNTs, and poly(3,4-ethylenedioxythiophene) (PEDOT) was fabricated via an electrochemical deposition strategy. This composite, when deposited onto a glassy carbon electrode (GCE), enabled the simultaneous electrochemical detection of dopamine (DA), serotonin (5-HT), and tryptophan (Trp). The presence of distinct oxidation peaks and linear responses for the analytes in micromolar ranges, with detection limits of 0.026 µM (DA), 0.063 µM (5-HT), and 0.210 µM (Trp), demonstrated the composite’s strong electrocatalytic activity due to the synergistic effects of the three components [16]. Atomic layer deposition (ALD) was utilized to uniformly anchor NiO nanoparticles (∼4.9 nm) onto CNT walls, creating a nanocomposite for the detection of hydroquinone (HQ) and catechol (CC). The resulting NiO/CNT-modified GCE exhibited enhanced redox peak separation and current responses. Detection limits were reported at 2.5 µM for both analytes, with linear ranges from 10 to 500 µM for HQ and 10–400 µM for CC, validating the nanocomposite’s application for environmental monitoring [85]. NiO was coated onto stacked-cut CNTs via ALD with varying cycles, and the resulting composites were evaluated for lactate sensing using a screen-printed carbon electrode configuration. Electrochemical measurements showed that the sample with 200 ALD cycles had optimal performance, delivering a sensitivity of 138.44 µA·mM^−1^·cm^−2^ and a low detection limit of 67 µM. The sensor demonstrated excellent analytical performance in human saliva, offering a non-invasive platform for lactate monitoring [86]. The probable mechanism for LA detection is shown in Figure 2.

A NiO–MWCNT nanocomposite-modified carbon paste electrode was also developed for nitrite detection. Morphological and compositional analyses confirmed the composite’s uniform structure, while electrochemical assessments (CV, EIS, and chronoamperometry) indicated a wide linear detection range (1–100 µM), high sensitivity (3.53 µA·µM^−1^), and low detection limit (0.25 µM). The sensor demonstrated excellent analytical performance in real tap water samples [87]. A comparative study of NiO, CuO, and Co_3_O_4_ nanoparticles supported on CNTs revealed their efficiency in glucose oxidation. Among them, Co_3_O_4_/CNTs exhibited the highest sensitivity and selectivity with a detection limit of 2 µM. Electrolysis product analysis by HPLC identified multiple glucose oxidation products, including glucuronic and formic acids, with Co_3_O_4_/CNTs showing the highest glucuronic acid selectivity (46.8%). These composites also achieved substantial glucose conversion rates, indicating their utility in catalytic glucose conversion and sensing [88]. Electrodeposited NiO@CNT composites on GCEs were evaluated for the electrochemical detection of fentanyl, a synthetic opioid. SEM and XRD analyses revealed a homogeneous core–shell structure with large void spaces, while electrochemical studies confirmed enhanced electron transfer kinetics. The sensor achieved a detection limit of 0.01 µM and a sensitivity of 1.12 µA/µM over a linear range of 10–160 µM. Comparative analyses indicated that the NiO@CNT/GCE platform outperformed existing fentanyl sensors in both range and sensitivity, validating its application in pharmaceutical and clinical sample analysis [89]. A highly sensitive and selective enzyme-free glucose sensor was constructed using NiO/MWCNT composites. The NiO nanoparticles synthesized via a precipitation-calcination method were uniformly immobilized onto MWCNTs, leading to improved surface area and charge transport. Electrochemical analysis revealed a broad linear range (0.5 µM to 3 mM), ultra-low LOD (~0.17 µM), high sensitivity (484 µA·mM^−1^·cm^−2^), and fast response time (~5 s). Computational modeling provided insights into glucose adsorption and oxidation mechanisms, confirming the synergistic role of NiO as an active redox site and MWCNTs as efficient charge mediators. The sensor exhibited high accuracy in salivary glucose determination, supporting its applicability for non-invasive diabetes monitoring [90]. A ternary nanohybrid of NiO@CNTs wrapped with graphene oxide (GO) was synthesized via a hydrothermal approach and utilized for dual detection of ascorbic acid (AA) and uric acid (UA). The electrochemical sensor showed distinct and well-separated redox peaks for AA and UA, with detection limits of 0.17 mM and 0.06 mM, respectively. The sensor displayed high sensitivity (14.62 µA/mM for AA and 15.61 µA/mM for UA), indicating potential for real-time biological sensing applications [91].

### 3.3. NiO/g-CN

**Figure 3:**
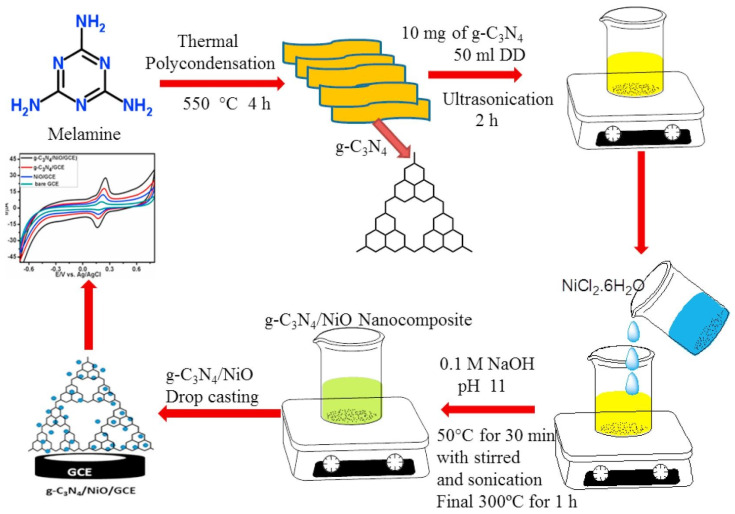
Schematic representation of the synthesis of g-C_3_N_4_/NiO composite and its application for electrochemical sensing. Reprinted with permission [95].

Graphitic carbon nitride (g-C_3_N_4_), owing to its unique electronic structure, excellent chemical stability, and facile synthesis, has emerged as a promising candidate for the development of electrochemical sensors. The integration of g-C_3_N_4_ with metal oxides such as nickel oxide (NiO) further enhances its surface area, charge transfer ability, and catalytic efficiency. This section presents a comprehensive overview of recent advancements in g-C_3_N_4_/NiO-based electrochemical sensors, focusing on their applications in the sensitive and selective detection of various drugs and biomolecules. A highly sensitive sensor for acetaminophen (AAP), an antipyretic and analgesic agent, was developed using a silver-decorated NiO/g-C_3_N_4_ nanocomposite synthesized via a sol–gel method followed by ultrasonic treatment. The modified glassy carbon electrode (Ag@NiO/g-C_3_N_4_/GCE) exhibited superior electrocatalytic activity, as demonstrated by cyclic voltammetry (CV) and differential pulse voltammetry (DPV) in phosphate-buffered saline (PBS, pH 7.0). The sensor displayed a linear detection range of 10 µM to 1 mM and a detection limit of 0.76 µM. High selectivity against interfering substances such as glucose, uric acid, and common salts affirmed its practical applicability [92]. Hydrogen peroxide (H_2_O_2_), extensively used in industrial and healthcare sectors, poses toxicity risks at elevated concentrations. A hydrothermal method followed by calcination at various temperatures (350–450 °C) was used to fabricate g-C_3_N_4_/NiO composites (CNNi-1, CNNi-2, CNNi-3). CNNi-2, exhibiting optimal hierarchical morphology and mesoporosity, was deposited on fluorine-doped tin oxide (FTO) substrates via spray pyrolysis. The electrode showed excellent electrochemical performance in H_2_O_2_ detection, with a broad linear range (0.001–0.750 μM), high sensitivity, and a low detection limit (0.10 ± 0.04 μM). The sensor-maintained stability over 8 weeks and demonstrated reproducibility (RSD = 3.23%) and practical utility in biological and environmental samples [93]. In another report, NiO/g-C_3_N_4_ hybrids, synthesized hydrothermally using melamine and Ni(OH)_2_ precursors, were explored for glucose detection applications. The optimal composition demonstrated exceptional glucose sensing sensitivity (5387.1 µA mM^−1^cm^−2^). A flexible, self-powered glucose sensor was also developed, integrating the same nanocomposite as sensing unit, suggesting potential for wearable diagnostic systems [94]. A one-pot synthesis approach yielded a g-C_3_N_4_/NiO nanocomposite for quercetin (QR) detection. The synthesis of the proposed material has been illustrated in Figure 3.

The electrochemical behavior was assessed via CV and DPV, revealing a rapid and linear current response over a wide QR concentration range (0.01–250 μM) with a low detection limit of 0.002 μM. Real sample analysis in green tea and fruits confirmed its suitability for practical applications [95]. NiO–Ni–graphitic carbon nitride (NiO–Ni–GCN) nanocomposites were synthesized via thermal treatment of melamine with NiCl_2_·6H_2_O. These materials demonstrated high electrocatalytic activity for the oxidation of octylphenol, an endocrine-disrupting compound. A DPV-based detection method under infrared light irradiation showed linearity across two concentration ranges (10 nM–1 μM and 1–50 μM) with a detection limit of 3.3 nM, validating the sensor’s environmental monitoring potential [96]. A hydrothermal method followed by annealing was employed to synthesize 3D hierarchical NiO/g-C_3_N_4_ composites. Characterization revealed a flower-like morphology and enhanced surface area. These composites (especially Ni/CN-10) exhibited a gas response of 20.03 to 500 ppm triethylamine (TEA), nearly triple that of pure NiO. The sensing enhancement was attributed to the formation of p–n heterojunctions and improved surface reactivity [97]. A photoelectrochemical (PEC) aptasensor based on NiO-decorated g-C_3_N_4_ was developed for the detection of *Staphylococcus aureus*. The DNA aptamer immobilized on the PEC electrode facilitated specific recognition, and bacterial binding caused a measurable signal suppression due to charge transfer inhibition. The sensor achieved high selectivity and sensitivity with a detection limit of 24 CFU/mL, suggesting its promise in point-of-care diagnostic applications [98]. A ternary g-C_3_N_4_/NiO/CuO nanocomposite was synthesized and immobilized on a GCE for glucose detection. The material was extensively characterized (FE-SEM, TEM, EDX, BET, XRD), and electrochemical studies confirmed superior electrocatalytic activity compared to binary counterparts. The sensor exhibited a wide linear range (0.4 µM–8.5 mM), a low detection limit (0.1 µM), and high sensitivity (362.12 µA mM^−1^ cm^−2^), along with good reproducibility and long-term performance [99].

### 3.4. NiO/MOF

Traditional analytical techniques for detecting heavy metals, though accurate, often require skilled personnel, lengthy procedures, and sophisticated instrumentation. To address these challenges, a portable electrochemical sensor was developed for the simultaneous detection of Pb^2+^ and Cu^2+^ in aqueous media. The sensor employed a nanocomposite comprising poly(amidoamine) dendrimers integrated with nickel-based metal–organic frameworks (PAMAM/Ni-MOFs) as a functional layer on a glassy carbon electrode (GCE). Detection was performed using square wave anodic stripping voltammetry (SWASV), revealing linear response ranges from 1 to 100 μg/L for both metal ions. The detection limits achieved (Pb^2+^: 1.21 μg/L; Cu^2+^: 0.77 μg/L) were well below the World Health Organization’s limits for potable water. The sensor demonstrated excellent selectivity (ΔI < 17% in presence of tenfold interferents), reproducibility (RSD < 3.56%), and stability (>95% signal retention after 30 days), making it a promising tool for real-time, on-site water quality assessment [7]. A multifunctional electrochemical sensing platform was fabricated by integrating Ni and NiO nanoparticles with a conductive carbon scaffold and Ni-MOF using a single-step calcination approach. The resulting Ni-MOF/Ni/NiO/carbon (C) nanocomposite was immobilized on a GCE with Nafion to create sensors capable of nonenzymatic detection of glucose and H_2_O_2_. The modified electrode exhibited enhanced electrocatalytic activity, with a sensitivity of 367.45 mA·M^−1^·cm^−2^, a low detection limit of 0.8 μM, and a broad linear range (4–5664 μM). The sensor offered stable, selective, and reproducible measurements, and its applicability was validated using real serum samples, highlighting the potential of MOF-derived NiO systems for biomedical sensing [100]. A novel glucose sensor was developed using a hierarchical NiO architecture grown on nickel foam. Ni-MOFs were first synthesized on Ni foam using a dual-metal source strategy and subsequently converted to NiO via pyrolysis. Morphological and structural analyses confirmed the formation of flake-like superstructures embedded in a three-dimensional conductive carbon network. Electrochemical tests demonstrated the sensor’s high sensitivity (395 μA·mM^−1^), low LOD (6.15 μM), and linear range of 0.018–1.2 mM, establishing it as a viable candidate for routine glucose analysis [101]. Calcination of polyaniline-coated Ni-MOFs yielded carbon- and nitrogen-doped NiO (CN-NiO) nanocomposites with flower-like morphology. These were used to modify GCEs for nonenzymatic glucose detection. The sensor exhibited a broad linear response (5 × 10^−7^ to 3 × 10^−3^ mol/L), a high sensitivity of 1144 μA·mM^−1^·cm^−2^, and an ultra-low detection limit of 0.5 μM. Importantly, the device demonstrated robust selectivity against common interferents such as urea, ascorbic acid, and dopamine, suggesting excellent promise for practical biosensing applications [102]. A pioneering application of conductive metal–organic frameworks in nitric oxide (NO) detection involved thin-film electrodes based on Ni_3_(HHTP)_2_ and other transition metal analogs. Among the tested MOFs, Ni- and Cu-based systems demonstrated the highest signal enhancements compared to bare GCEs. When coupled with a conductive polymer, the Ni-MOF electrode achieved stable amperometric detection of NO at nanomolar concentrations (LOD: 9.0 ± 4.8 nM), along with moderate selectivity over ascorbic acid and nitrite [103]. A composite of NiO and ZnO hollow microspheres was synthesized through the thermal decomposition of Zn^2+^/Ni^2+^-based MOFs. These microspheres, with porous surface structures, demonstrated enhanced conductivity and catalytic activity toward isoniazid oxidation. Electrochemical evaluation using cyclic and differential pulse voltammetry revealed a wide detection range (0.8–800 μM) and a low LOD (0.25 μM). The sensor-maintained reproducibility and accuracy across pharmaceutical and biological matrices, supporting its utility for drug monitoring [104]. A 3D sensor interface was constructed by sequentially growing NiO nanoarrays and Ni-MOF layers on titanium mesh (TM). The synergistic combination of high surface area (from Ni-MOF), catalytic efficiency (from NiO), and conductivity (from TM) led to exceptional sensing capabilities for the flavonoid luteolin. The platform achieved a detection limit as low as 3 pM and displayed excellent selectivity and real-sample applicability, establishing it as a powerful tool for natural product analysis [105]. A porous nanocomposite composed of Ni-MOF, NiO, and conductive carbon was synthesized at 450 °C and used as a nonenzymatic sensor for glutamate. The material showed high electrocatalytic activity, with a detection range of 5–960 μmol/L and a LOD of 320 nmol/L. When validated against HPLC analysis, the sensor exhibited comparable results with high recovery, accuracy, and repeatability, confirming its practical suitability for clinical and food-related applications [106]. A ternary nanocomposite of Cu-MOF, CuO, and NiO was fabricated via a hydrothermal route using Cu-MOF as a sacrificial template. The hybrid sensor demonstrated superior electrochemical properties due to enhanced catalytic activity, high surface area, and synergistic effects among the metal oxides. The sensor achieved a low LOD (0.0078 µM) and LOQ (0.026 µM), with a broad linear range (0.01–22 µM) for catechin detection. Successful application to tea samples confirmed its relevance for real-world food analysis and hinted at broader applications in sensing and catalysis [107].

### 3.5. NiO/Zeolitic Imidazolate Framework (ZIF)

**Figure 4:**
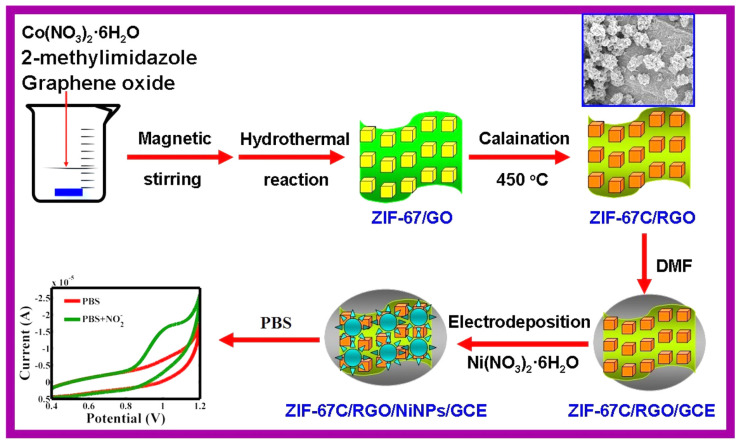
Schematic representation of the preparation of ZIF-67C/RGO and fabrication of ZIF-67C/RGO/NiNPs/GCE. Reprinted with permission [109].

A non-enzymatic glucose sensor was constructed from 2D NiO/Co_3_O_4_@C heterojunctions obtained by pyrolyzing NiO@ZIF-67. The Nafion-modified sensor exhibited wide linear detection ranges (5–1000 μM and 1–4 mM) and high sensitivities (690 and 215.4 μA·mM^−1^·cm^−2^), with good recovery in serum samples and excellent reproducibility [108]. We have also included Ni NPs based composite with ZIF and RGO for the determination of nitrite as shown in Figure 4 [109]. The carbonized ZIF-67 (ZIF-67)/RGO/ Ni NPs modified electrode was used as nitrite sensor.

The modified electrode showed enhanced amperometric responses with a dual linear range (0.2–123 μM and 123–473 μM) and a detection limit of 0.086 μM. This sensor demonstrated high stability and anti-interference capacity, making it suitable for food safety applications. This is showing that presence of Ni NPs enhance the detection of nitrite. Thus, it is clear that presence of Ni in the NiO improve the catalytic properties of NiO. A C-ZIF-8/NiO composite was fabricated as a photocathode, leveraging improved light absorption and electron transfer. Hemin-sensitized photoelectrochemical measurements allowed ultrasensitive detection of lead ions with a linear range of 10 pM–5 nM and a detection limit as low as 2.6 pM [110]. A multifunctional sensor comprising Ni-ZIF-8, N,S-doped CNTs, and chitosan (CS) was developed for concurrent detection of dopamine, uric acid, and L-tryptophan. The sensor showed excellent signal resolution and linear detection ranges for each analyte, with LODs of 0.93, 0.41, and 0.69 μM, respectively, making it suitable for biological sample analysis [111]. A poly(caffeic acid) film combined with Zn/Ni-ZIF-8-derived carbon was used for acetaminophen (AP) sensing. The hybrid material offered high conductivity and catalytic activity, yielding a low detection limit (0.0291 μM) and successful application in pharmaceutical and biological samples [112]. An electrochemical sensor based on Co_3_O_4_-LaMn_0.5_Ni_0.5_O_3_/MWCNTs derived from ZIF-67 was engineered for the dual detection of protocatechuic acid (PCA) and ascorbic acid (AA). The sensor achieved high resolution and linearity in the oxidation peaks, with detection limits of 8.83 and 7.35 μM, respectively, and was successfully applied to real beverage samples [113].

### 3.6. NiO/COF

Recent advancements in covalent organic framework (COF)-based composites have enabled the development of highly sensitive, enzyme-free electrochemical sensors. A Ni/NiO-embedded nitrogen-doped carbon (Ni/NiO/NC) material was synthesized via a templated strategy involving the growth of a COF with O- and N-rich chelating sites on a SiO_2_ scaffold, coordination with Ni^2+^ ions, followed by carbonization and template removal. The resultant Ni/NiO/NC nanocomposite exhibited tunable electrocatalytic activity governed by the pore structure and nanoparticle size. This material demonstrated excellent glucose sensing performance, with a detection limit of 0.2 μM, a broad linear range (0.6 μM–8.6 mM), and a high sensitivity of 76.03 μA·mM^−1^·cm^−2^. Additionally, a wearable, paper-based sensor utilizing the same composite achieved a detection limit of 0.4 μM and maintained good sensitivity (42.38 μA·mM^−1^·cm^−2^) across relevant glucose concentrations. These sensors exhibited high selectivity and practical applicability in real matrices such as fruit juice, human serum, and sweat [114].

### 3.7. NiO/Mxene

The pursuit of efficient, sensitive, and scalable photoelectrochemical (PEC) and electrochemical sensors has led to the development of innovative nanocomposites, particularly those incorporating NiO and MXenes. These materials have demonstrated significant potential in non-enzymatic glucose detection, neurotransmitter sensing, pH monitoring, and biomarker identification due to their exceptional electrochemical activity, structural versatility, and surface functionalization. A novel ternary composite, MXene@NiO-ZnO (NMZ), was synthesized using a dual-pressure hydrothermal approach that integrates simultaneous pressure regulation and active stirring. This technique ensures the uniform co-precipitation of Ni^2+^ and Zn^2+^ ions with carbonate and hydroxyl anions on the MXene substrate. The resulting composite exhibits a hierarchical porous structure with a large surface area (72.4 m^2^/g) and a significantly enhanced electrochemical active area (2 cm^2^). The internal electric field at the ZnO/NiO interface facilitates effective separation of photogenerated charges, while MXene incorporation mitigates Schottky barriers, promoting unhindered electron transport to the electrode surface. Additionally, NiO’s favorable valence band potential (2.45 eV vs. NHE) drives hydroxyl radical formation and Ni(OH)_2_ generation, key for glucose oxidation. The NMZ-based PEC glucose sensor delivers remarkable performance, achieving high sensitivity (7651.2 μA mM^−1^ cm^−2^), a rapid response time (4 s), and excellent reproducibility (RSD of 5.21%), outperforming binary ZnO/NiO systems [115]. A ternary nanohybrid comprising NiO-reduced graphene oxide (rGO)/Mo_2_Ti_2_C_3_ MXene was fabricated through an in situ synthesis route, incorporating electrochemically active rGO derived from industrial graphite waste. The synergistic combination of MXene and rGO facilitates enhanced charge transport and active site availability, making the material a potent electrocatalyst for dopamine oxidation. The modified electrode (NGM21) exhibited high sensitivity (1.441 × 10^−4^ μA μM^−^^1^), a broad detection range (2–12 nM), and a low detection limit of 1.44 nM. Differential pulse voltammetry (DPV) and cyclic voltammetry (CV) showed excellent selectivity and minimal interference from coexisting species. The sensor’s applicability in real samples was validated with recovery rates of 98–107% in serum and urine, indicating its promise for clinical diagnostics [17]. In efforts to develop non-invasive glucose sensors, a composite consisting of NiO nanoparticles decorated polyaniline nanosheets (PANI NS) anchored on Ti_3_C_2_T_x_ MXene (NiOMP) was synthesized via an ice-bath-assisted ultrasonication process. This method ensured homogeneous dispersion of NiO NPs on the conductive polymer-MXene matrix. Morphological analysis using FESEM and TEM revealed uniform nanoparticle distribution. Electrochemical evaluation using screen-printed carbon electrodes (SPCE) showed excellent sensitivity (3551.53 μA mM^−1^ cm^−2^), a low detection limit of 0.019 μM, and a dynamic linear range of 5–500 μM. The sensor demonstrated high selectivity and reproducibility when tested on sweat samples from diabetic volunteers, proving its potential for wearable glucose monitoring devices [116]. Accurate and reliable pH sensing is vital across diverse industries such as agriculture, healthcare, and environmental monitoring. A multifunctional NiO/MXene/PANI composite was synthesized and integrated onto a nickel foam (NF) electrode to develop a solid-state pH sensor. NiO nanoparticles were first synthesized via a sol–gel route, followed by successive incorporation of MXene and PANI. The composite was electrodeposited onto the NF substrate. Electrochemical performance was evaluated using CV, amperometry, and open circuit potential (OCP) methods. MXene’s surface groups (–OH, –O) contributed to its high pH responsiveness via protonation/deprotonation in acidic or basic media. The sensor exhibited linear sensitivity of 46.00 mV/pH over a broad pH range (3–11) with excellent reproducibility (R^2^ = 0.99). Testing against real samples (e.g., citrus and baking powder solutions) demonstrated strong correlation with commercial pH meters, indicating this sensor’s practicality for diverse applications [117].

### 3.8. NiO/Polymers

The design of high-performance non-enzymatic electrochemical sensors utilizing nanostructured materials has gained significant momentum due to their superior sensitivity, selectivity, and eco-compatibility. In this context, ZnO/NiO nanocomposites embedded in a natural tragacanth gum matrix (ZNT NCs) have been synthesized via a green approach for the ultrasensitive detection of riboflavin (RF). Tragacanth gum, a naturally occurring biopolymer, provides a large surface area and biocompatibility, facilitating the stable dispersion of metal oxides. Electrochemical characterization on a glassy carbon electrode (GCE) modified with the ZNT NCs demonstrated enhanced conductivity (~1.6 × 10^−5^ A), as evaluated by cyclic voltammetry (CV) and electrochemical impedance spectroscopy (EIS) in a [Fe(CN)_6_]^3−^/^4−^ medium. Square wave voltammetry (SWV) under phosphate buffer conditions further confirmed increased electrocatalytic activity, attributed to an enlarged electroactive surface area (2.74 cm^2^). The developed sensor exhibited a low limit of quantification (LOQ) of 2.24 µM and remarkable sensitivity (1002.92 µA·µM^−1^·cm^−2^). A wide linear detection range (10 nM to 1100 µM) and a low detection limit (8.7 nM) were achieved due to electrostatic interactions between hydroxyl groups in the biopolymer and RF molecules [118]. Another significant advancement involves the use of polyaniline (PANI) in conjunction with metal oxide nanoparticles (NiO, ZnO, Fe_3_O_4_) to develop composite-modified GCEs for the electrochemical detection of dopamine (DA), even in the presence of common interferents such as ascorbic acid (AA) and serotonin (SE). Using differential pulse voltammetry (DPV) at physiological pH (7.0), the detection range spanned from 2.0 × 10^−5^ to 2.4 × 10^−6^ M. Among the variants, the PANI-NiO modified electrode showed superior performance with the lowest LOD of 1.53 × 10^−8^ M, suggesting its suitability for DA analysis in pharmaceutical preparations [18]. A calixarene-functionalized NiO nanomaterial (p-HNC6/NiO) has also been engineered for the selective detection of bisphenol S (BPS). The material was drop-cast onto GCE, forming a stable sensing interface. Under optimized conditions (Britton–Robinson buffer, pH 4), the sensor displayed excellent electrochemical response with a linear range of 0.8–70 µM, a low LOD (5.9 nM), and LOQ (19 nM), making it a viable candidate for real-world wastewater analysis [119]. In another study, a composite material comprising Au@NiO nanoparticles embedded in a polypyrrole (PPy) matrix was developed as a non-enzymatic cholesterol sensor. The composite-modified GCE exhibited a broad linear detection range (1.0 × 10^−5^ to 1.0 × 10^−4^ M), high sensitivity (7.6 µA·µM^−1^·cm^−2^), and a low detection limit (5.8 × 10^−7^ M), indicating its feasibility for clinical sensor applications [120]. For the detection of the organophosphorus pesticide malathion, a PANI-ZnO–NiO nanocomposite-modified electrode was fabricated. The sensor exhibited excellent performance with a linear response in the 10–70 nM range and a detection limit of 1.0 × 10^−8^ M, demonstrating its high sensitivity and practical relevance for environmental monitoring [121]. Nickel and copper oxide nanoparticles incorporated into polyaniline nanofibers (NiO/CuO/PANI) have been explored for non-enzymatic glucose sensing. The sensor showed a wide linear range (20–2500 µM), a detection limit of 2.0 µM, and high selectivity in serum samples, highlighting its potential in biomedical diagnostics [22]. Furthermore, a co-polymer composite comprising polyaniline and polypyrrole doped with varying wt% of NiO [Poly(Ani-co-Py)/NiO] was synthesized via oxidative polymerization and evaluated for its dual functionality: photocatalytic degradation of methylene blue and electrochemical sensing of p-nitrophenol (p-NP). The sensor probe, based on Poly(Ani-Co-Py)/NiO integrated with PEDOT:PSS and deposited on GCE, exhibited high sensitivity (12.658 µA·µM^−1^·cm^−2^), a broad linear range (0.1–80.0 µM), and a low LOD (0.075 µM), thus presenting a promising strategy for detecting environmental contaminants [122].

### 3.9. NiO/LDH

**Figure 5:**
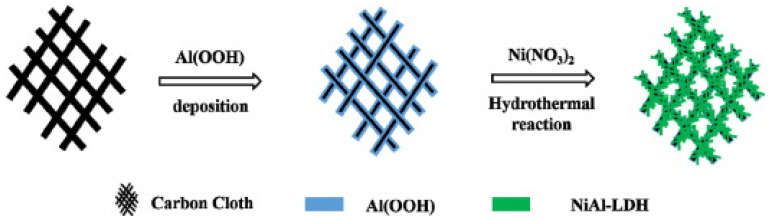
Schematic representation of the fabrication of NiAl-LDH using hydrothermal method on carbon cloth. Reprinted with permission [125].

Layered double hydroxides (LDHs), known for their hydrotalcite-like layered structures and tunable compositions, have emerged as versatile materials in the design of high-performance electrochemical sensors. Their unique properties—including large surface area, redox-active metal centers, and structural flexibility—make them suitable for the detection of a wide range of analytes, including biologically relevant molecules, pesticides, and pharmaceutical compounds. Epicatechin (EC), a polyphenolic compound prevalent in tea, plays a pivotal role in human health owing to its antioxidant properties. However, fluctuations in EC levels are associated with the onset of chronic disorders, necessitating its accurate and sensitive detection. In a recent study, a nickel–iron layered double hydroxide (NiFe-LDH) was fabricated and employed for the electrochemical sensing of EC. The sensing mechanism is primarily governed by electrostatic interactions between metal ions in the LDH and the hydroxyl functionalities of EC. The sensor exhibited two linear response ranges (50 nM–1 μM and 10–400 μM), with detection limits of 9.54 nM and 14.2 μM, respectively. It demonstrated excellent cycling stability (87% after 150 cycles) and good reproducibility (RSD = 3.8%). Although selectivity was slightly impacted by structurally similar compounds like quercetin and epigallocatechin gallate, the sensor exhibited satisfactory performance in real tea samples, validating its potential as a viable electrochemical platform for EC detection [123]. Nickel–cobalt LDH hollow nanostructures were synthesized and utilized to develop a modified screen-printed electrode (SPE) for the sensitive detection of sumatriptan, a migraine medication. The modified electrode (Ni–Co LDH/SPE) demonstrated improved electron transfer kinetics and reduced oxidation overpotential of sumatriptan compared to the bare SPE. Using differential pulse voltammetry (DPV), the sensor exhibited a broad linear detection range (0.01–435 μM), a low detection limit (2 nM), and high sensitivity (0.1017 μA/μM). Importantly, the sensor achieved selective detection even in the presence of naproxen, a common pharmaceutical interferent, and was validated in pharmaceutical and biological matrices with high recovery rates [124]. A flexible, enzyme-free glucose sensor based on NiAl-LDH sheets grown in situ on carbon cloth (CC) was developed (Figure 5). The resulting 3D macro-porous structure enhanced electron/ion transfer, contributing to the sensor’s ultrahigh sensitivity (14.13 mA·mM^−1^·cm^−2^) and rapid response time (<1 s). Even half-sized electrodes maintained high performance, indicating robustness and scalability. These features render the NiAl-LDH/CC sensor highly promising for portable, low-cost glucose monitoring applications [125].

A hybrid NiCo-LDH/functionalized halloysite nanotubes (F-HNTs) composite was synthesized via hydrothermal synthesis and utilized for parathion (PT) detection. The synergistic interaction between the LDH layers and F-HNTs provided abundant active sites, improved conductivity, and rapid electron transfer. The sensor demonstrated excellent analytical figures of merit, including a wide linear detection range (0.01–33.4 µM), a low LOD (2 nM), and high sensitivity (13.0 µA·µM^−1^·cm^−2^). Moreover, the platform showed consistent performance in real environmental samples, highlighting its application for pesticide residue analysis [126]. To overcome limitations related to enzyme stability in lactate biosensors, Ni-based LDHs incorporating secondary metals (Fe, Co) were explored. The electrocatalytic behavior was evaluated using screen-printed carbon electrodes (SPCEs), with NiCo-LDH exhibiting the highest sensitivity (30.59 µA·mM^−1^·cm^−2^) and broader linear range (5–25 mM), outperforming NiFe and pristine Ni-LDHs. The enhanced activity was attributed to the Co centers that facilitated lactate oxidation and OH− adsorption, offering a promising route for stable, enzyme-free lactate monitoring in physiological conditions [127]. A highly sensitive, flexible, non-enzymatic glucose sensor was developed using a hierarchical nanostructure composed of NiCu-LDH grown on CuO nanorods supported by nitrogen-doped carbon cloth (NCC). The NiCu-LDH/CuO/NCC architecture provided enhanced surface area, active sites, and conductivity. Optimization studies revealed the best performance at a Ni/Cu molar ratio of 2:1, with the sensor demonstrating a sensitivity of 11.545 mA·mM^−1^·cm^−2^, a low detection limit (0.26 µM), and a wide linear range (1 µM–1.5 mM). The sensor maintained 95% of its activity over 28 days, highlighting its stability and practical potential in non-invasive glucose diagnostics [128]. The electrochemical detection of carbendazim (CDM), a widely used fungicide, was accomplished using a NiCo-LDH modified glassy carbon electrode. Structural and surface characterizations confirmed the integrity of the LDH. The sensor exhibited a significant anodic peak at +0.87 V vs. Ag/AgCl, with a broad dynamic range (0.006–14.1 µM), an exceptionally low LOD (1 nM), and sensitivity of 3.38 µA·µM^−1^·cm^−2^. Its performance in river water samples further underscored its applicability for environmental monitoring [129].

### 3.10. NiO Overview

Nickel oxide (NiO) has attracted considerable attention as an electrochemical sensing material due to its low cost, environmental benignity, and intrinsic redox activity. Despite these advantages, the practical implementation of NiO-based sensors is constrained by several inherent limitations. A major drawback is NiO is intrinsically poor electrical conductivity, stemming from its wide bandgap semiconductor characteristics (~3.6–4.0 eV), which impedes efficient electron transport and consequently diminishes sensor sensitivity and response rates. Furthermore, the tendency of NiO nanostructures to agglomerate during synthesis or operational cycles leads to a significant reduction in active surface area and available electroactive sites, thereby compromising overall sensing performance. Structural degradation under prolonged electrochemical cycling further limits the long-term operational stability of these materials. In addition, pristine NiO typically exhibits limited selectivity in complex environments, primarily due to the absence of specific molecular recognition sites, making it susceptible to interference from non-target electroactive species. Lastly, the sluggish ion diffusion and charge transfer kinetics inherent to NiO often result in delayed response and recovery times, posing further challenges for real-time sensing applications.

To address the aforementioned limitations, various modification strategies have been investigated to enhance the electrochemical performance of NiO-based sensors. The fabrication of NiO composites with highly conductive materials—such as graphene, carbon nanotubes (CNTs), and conductive polymers—has proven effective in significantly improving electrical conductivity, electron transport, and structural robustness. Moreover, the incorporation of molecular recognition elements, including aptamers and molecularly imprinted polymers, onto the NiO surface has been employed to enhance selectivity, particularly in complex sample matrices. Structural engineering approaches, such as nanostructuring and elemental doping with transition metals (e.g., cobalt, iron, or copper), have also been shown to modulate the electronic structure, increase active sites, and facilitate redox kinetics. Additionally, the integration of NiO-based materials into flexible and wearable sensor platforms is gaining traction, offering promising prospects for next-generation, real-time, and on-site monitoring applications.

## 4. Conclusions

Nickel oxide (NiO)-based materials have emerged as highly promising candidates for electrochemical sensing due to their advantageous physicochemical characteristics and continuous advancements in material design strategies. The development of nanostructured NiO and its hybrid composites with conductive, porous, and redox-active components has markedly enhanced key sensor performance parameters such as sensitivity, selectivity, and response time. Our main findings are as follows:

- NiO-modified electrodes have been extensively utilized for the electrochemical detection of biologically significant analytes, including glucose, dopamine, ascorbic acid, uric acid, and hydrogen peroxide.
- These sensing platforms have also shown strong potential for monitoring environmental contaminants such as heavy metal ions (e.g., Pb^2+^, Cd^2+^), pesticides, phenolic derivatives, and volatile organic compounds, offering a rapid, sensitive, and economically viable alternative to conventional analytical methods.
- In the food and pharmaceutical industries, NiO-based sensors have been effectively employed for the analysis of additives, preservatives, and active pharmaceutical ingredients, thereby supporting quality control and regulatory compliance.
- The integration of NiO-based sensing systems with emerging technologies such as machine learning, data analytics, and the Internet of Things (IoT) is expected to usher in a new era of intelligent, autonomous, and interconnected electrochemical sensing platforms for healthcare diagnostics and environmental monitoring.
- Despite substantial progress, challenges related to the long-term operational stability, reproducibility, and selectivity of these sensors, especially in complex real-world matrices, remain to be addressed.
- Furthermore, the incorporation of NiO-based sensors into miniaturized, flexible, and wearable devices represents a rapidly evolving research direction, driven by the need for portable and point-of-care diagnostic solutions.

Continued research focusing on novel composite architectures, surface functionalization strategies, and device-level integration is anticipated to further expand the utility of NiO-based electrochemical sensors and establish their significance in the broader domain of analytical and sensor technologies.

## Data Availability

Data sharing is not possible. No new data were generated.

## References

[1] Zhang L., Guo W., Lv C., Liu X., Yang M., Guo M., Fu Q. (2023). Electrochemical Biosensors Represent Promising Detection Tools in Medical Field. Adv. Sens. Energy Mater..

[2] Singh R., Gupta R., Bansal D., Bhateria R., Sharma M. (2024). A Review on Recent Trends and Future Developments in Electrochemical Sensing. ACS Omega.

[3] Lawal A.T. (2023). Recent Developments in Electrochemical Sensors Based on Graphene for Bioanalytical Applications. Sens. Bio-Sens. Res..

[4] Paulose R., Mohan R., Parihar V. (2017). Nanostructured Nickel Oxide and Its Electrochemical Behaviour—A Brief Review. Nano-Struct. Nano-Objects.

[5] Ahamed N.N., Pattar J., Murthy R.N.K., Kumar M.R.A., Bhoomika V., Raghavendra N., Ravikumar C.R. (2024). Electrochemical Studies of Nickel Oxide Nanoparticles via Solution Combustion Method Using Green and Chemical Fuels. Sustain. Chem. Environ..

[6] Ahamad N., Banerjee S., Wei C.-C., Lu K.-C., Khedulkar A.P., Jian W.-B., Mahmood S., Chu C.-W., Lin H.-C. (2024). Flexible Non-Enzymatic Glucose Sensors: One-Step Green Synthesis of NiO Nanoporous Films via an Electro-Exploding Wire Technique. ACS Appl. Mater. Interfaces.

[7] Wang M., Chen Y., Noureen B., Ma Y., Zheng A., Zhou L., Liu M., Zhao Y., Du L., Zhang W. (2025). An Electrochemical Sensor Using Nickel-Based Metal-Organic Framework towards Highly-Sensitive Detection of Multiple Heavy Metal Ions. Sens. Actuators B Chem..

[8] Mondal R., Ahmed S.F., Mukherjee N. (2025). Highly Sensitive, Selective and Rapid in-Vitro Electrochemical Sensing of Dopamine Achieved on Oxygen Deficient Nickel Oxide/Partially Reduced Graphene Oxide (NiO_x_/p-rGO) Nanocomposite Platform. Inorg. Chem. Commun..

[9] Prasad R., Bhat B.R. (2015). Multi-Wall Carbon Nanotube–NiO Nanoparticle Composite as Enzyme-Free Electrochemical Glucose Sensor. Sens. Actuators B Chem..

[10] Ahmad R., Shah M.A. (2023). Hydrothermally Synthesised Nickel Oxide Nanostructures on Nickel Foam and Nickel Foil for Supercapacitor Application. Ceram. Int..

[11] Chatzichristidi M., Psycharis V., Katsoufis P., Stavridis N., Kontoliou A., Makarona E. (2025). Facile and Rapid Microwave Synthesis of Nickel Oxide Nanoparticles towards Cost-Efficient Functional Nanocomposite Photoresists. ChemistrySelect.

[12] Nkele A.C., Alshoaibi A., Matthew F.D., Awada C., Islam S., Ezema F.I. (2025). Synthesis and Characterization of Sol-Gel Processed GO/NiO Hybrid Composites for Gas Sensing and Photocatalytic Applications. J. Sol-Gel Sci. Technol..

[13] Wilson R.L., Macdonald T.J., Lin C.-T., Xu S., Taylor A., Knapp C.E., Guldin S., McLachlan M.A., Carmalt C.J., Blackman C.S. (2021). Chemical Vapour Deposition (CVD) of Nickel Oxide Using the Novel Nickel Dialkylaminoalkoxide Precursor [Ni(Dmamp′)_2_] (Dmamp′ = 2-Dimethylamino-2-Methyl-1-Propanolate). RSC Adv..

[14] Ben Arbia M., Comini E. (2024). Growth Processing and Strategies: A Way to Improve the Gas Sensing Performance of Nickel Oxide-Based Devices. Chemosensors.

[15] Rengaraj A., Haldorai Y., Kwak C.H., Ahn S., Jeon K.-J., Park S.H., Han Y.-K., Huh Y.S. (2015). Electrodeposition of Flower-like Nickel Oxide on CVD-Grown Graphene to Develop an Electrochemical Non-Enzymatic Biosensor. J. Mater. Chem. B.

[16] Sun D., Li H., Li M., Li C., Dai H., Sun D., Yang B. (2018). Electrodeposition Synthesis of a NiO/CNT/PEDOT Composite for Simultaneous Detection of Dopamine, Serotonin, and Tryptophan. Sens. Actuators B Chem..

[17] Sahadevan K., Vinoba M., Revathi S., Jeong S.K., Bhagiyalakshmi M. (2025). Facile Design of NiO-rGO/Mo2Ti2C3 Ternary Composites for Electrochemical Detection of Dopamine. Synth. Met..

[18] Fayemi O.E., Adekunle A.S., Kumara Swamy B.E., Ebenso E.E. (2018). Electrochemical Sensor for the Detection of Dopamine in Real Samples Using Polyaniline/NiO, ZnO, and Fe_3_O_4_ Nanocomposites on Glassy Carbon Electrode. J. Electroanal. Chem..

[19] Yin Y., Zhu Y., Qian L., Wang F., Yuan Z., Dai Y., Zhang T., Yang D., Qiu F. (2025). Modulation of Electronic Structure Caused by the Coupling between Al, P Modified NiO and Ru for Boosting Alkaline Hydrogen Evolution. Fuel.

[20] Azami M., Wei J., Valizadehderakhshan M., Jayapalan A., Ayodele O.O., Nowlin K. (2023). Effect of Doping Heteroatoms on the Optical Behaviors and Radical Scavenging Properties of Carbon Nanodots. J. Phys. Chem. C.

[21] Nunes W.G., Miranda A.N., Freitas B., Vicentini R., Oliveira A.C., Doubek G., Freitas R.G., Silva L.M.D., Zanin H. (2021). Charge-Storage Mechanism of Highly Defective NiO Nanostructures on Carbon Nanofibers in Electrochemical Supercapacitors. Nanoscale.

[22] Ghanbari K., Babaei Z. (2016). Fabrication and Characterization of Non-Enzymatic Glucose Sensor Based on Ternary NiO/CuO/Polyaniline Nanocomposite. Anal. Biochem..

[23] Khashaei M., Kafi-Ahmadi L., Khademinia S., Poursattar Marjani A., Nozad E. (2022). A Facile Hydrothermal Synthesis of High-Efficient NiO Nanocatalyst for Preparation of 3,4-Dihydropyrimidin-2(1H)-Ones. Sci. Rep..

[24] Dudorova D.A., Simonenko T.L., Simonenko N.P., Gorobtsov P.Y., Volkov I.A., Simonenko E.P., Kuznetsov N.T. (2023). Hydrothermal Synthesis of Nickel Oxide and Its Application in the Additive Manufacturing of Planar Nanostructures. Molecules.

[25] Islam M.R., Bhuiyan M.A., Ahmed M.H., Rahaman M. (2024). Hydrothermal Synthesis of NiO Nanoparticles Decorated Hierarchical MnO2 Nanowire for Supercapacitor Electrode with Improved Electrochemical Performance. Heliyon.

[26] Kesmez Ö., Kuruca T., Odabaş E., Cihanoğlu N., Akarsu E., Demir F. (2025). Synthesis and Characterization of Nickel Oxide Nanoparticles via Polyol-Mediated Hydrothermal Process with Antibacterial Properties. ChemistrySelect.

[27] Qian G., Peng Q., Zou D., Wang S., Yan B. (2020). Hydrothermal Synthesis of Flake-Flower NiO and Its Gas Sensing Performance to CO. Front. Mater..

[28] Cao S., Zeng W., Li T., Gong J., Zhu Z. (2015). Hydrothermal Synthesis of NiO Nanobelts and the Effect of Sodium Oxalate. Mater. Lett..

[29] Babu G.A., Ravi G., Mahalingam T., Kumaresavanji M., Hayakawa Y. (2015). Influence of Microwave Power on the Preparation of NiO Nanoflakes for Enhanced Magnetic and Supercapacitor Applications. Dalton Trans..

[30] Parada C., Morán E. (2006). Microwave-Assisted Synthesis and Magnetic Study of Nanosized Ni/NiO Materials. Chem. Mater..

[31] Miah M.Y., Halder S., Saikat S.P., Dewanjee S., Ashaduzzaman M., Bhowmik S. (2025). Microwave-Assisted One-Step Synthesis of Polyacrylamide/NiO Nanocomposite for Biomedical Applications. RSC Adv..

[32] Tohidiyan Z., Hashemi S., Boroujeni K.P. (2019). Facile Microwave-Assisted Synthesis of NiO Nanoparticles and Its Effect on Soybean (*Glycine max*). IET Nanobiotechnol..

[33] Azhagu Raj R., AlSalhi M.S., Devanesan S. (2017). Microwave-Assisted Synthesis of Nickel Oxide Nanoparticles Using Coriandrum Sativum Leaf Extract and Their Structural-Magnetic Catalytic Properties. Materials.

[34] Meher S.K., Justin P., Ranga Rao G. (2011). Microwave-Mediated Synthesis for Improved Morphology and Pseudocapacitance Performance of Nickel Oxide. ACS Appl. Mater. Interfaces.

[35] Pilban Jahromi S., Huang N.M., Muhamad M.R., Lim H.N. (2013). Green Gelatine-Assisted Sol–Gel Synthesis of Ultrasmall Nickel Oxide Nanoparticles. Ceram. Int..

[36] Yang Q., Sha J., Ma X., Yang D. (2005). Synthesis of NiO Nanowires by a Sol-Gel Process. Mater. Lett..

[37] El-Katori E.E., Kasim E.A., Ali D.A. (2022). Sol–Gel Synthesis of Mesoporous NiO/ZnO Heterostructure Nanocomposite for Photocatalytic and Anticorrosive Applications in Aqueous Media. Colloids Surf. A Physicochem. Eng. Asp..

[38] Shen Y., Lua A.C. (2014). Sol–Gel Synthesis of Ni and Ni Supported Catalysts for Hydrogen Production by Methane Decomposition. RSC Adv..

[39] Zorkipli N.N.M., Kaus N.H.M., Mohamad A.A. (2016). Synthesis of NiO Nanoparticles through Sol-Gel Method. Procedia Chem..

[40] Dhas S.D., Maldar P.S., Patil M.D., Waikar M.R., Sonkawade R.G., Moholkar A.V. (2022). Sol-Gel Synthesized Nickel Oxide Nanostructures on Nickel Foam and Nickel Mesh for a Targeted Energy Storage Application. J. Energy Storage.

[41] Mateos D., Valdez B., Castillo J.R., Nedev N., Curiel M., Perez O., Arias A., Tiznado H. (2019). Synthesis of High Purity Nickel Oxide by a Modified Sol-Gel Method. Ceram. Int..

[42] Gidey A.T., Kuo D.-W., Fenta A.D., Chen C.-T., Chen C.-T. (2021). First Conventional Solution Sol–Gel-Prepared Nanoporous Materials of Nickel Oxide for Efficiency Enhancing and Stability Extending MAPbI3 Inverted Perovskite Solar Cells. ACS Appl. Energy Mater..

[43] Salleh N.A., Mohammad A.H., Zakaria Z., Deghfel B., Yaakob M.K., Rahiman W., Kheawhom S., Mohamad A.A. (2024). Microwave Assisted Synthesis of Nickel Oxide Nanoparticles at Different pH via Sol Gel Method: Experimental and First-Principles Investigations. Inorg. Chem. Commun..

[44] Alam M.A., Ahmed S., Bishwas R.K., Sarkar D., Jahan S.A. (2025). Crystal Growth Behavior Interpret of Co-Precipitated Derived Nickel Oxide (NiO) Nanocrystals. Nano-Struct. Nano-Objects.

[45] Godlaveeti S.K., Rajesh N., Ouladsmane M., Aljuwayid A.M., Riazunnisa K., Azharuddin S.M., Chintaparty R. (2025). Structural, Optical, and Antibacterial Properties of NiO and BaO Doped NiO- Prepared by Co-Precipitation Method. BioNanoScience.

[46] Atul A.K., Srivastava S.K., Gupta A.K., Srivastava N. (2021). Synthesis and Characterization of NiO Nanoparticles by Chemical Co-Precipitation Method: An Easy and Cost-Effective Approach. Braz. J. Phys..

[47] Al Boukhari J., Bitar Z., Azab A.A., Awad R., Rekaby M. (2023). Synthesis and Physical Properties of Pure NiO and Ni_1–2x_Mg_x_M_x_O (M= Cu, Ru) Nanoparticles: Role of Growth Temperature. J. Alloys Compd..

[48] Li J., Luo F., Zhao Q., Li Z., Yuan H., Xiao D. (2014). Coprecipitation Fabrication and Electrochemical Performances of Coral-like Mesoporous NiO Nanobars. J. Mater. Chem. A.

[49] Kaur N., Singh J., Kaur G., Kumar S., Kukkar D., Rawat M. (2019). CTAB Assisted Co-precipitation Synthesis of NiO Nanoparticles and Their Efficient Potential towards the Removal of Industrial Dyes. Micro Nano Lett..

[50] Lin H., Wang Z., Han Q., Wang R., Pan L., Zhu H., Wan M., Mai Y. (2021). The Growth, Properties and Application of Reactively Sputtered Nickel Oxide Thin Films in All Thin Film Electrochromic Devices. Mater. Sci. Eng. B.

[51] Mei B., Permyakova A.A., Frydendal R., Bae D., Pedersen T., Malacrida P., Hansen O., Stephens I.E.L., Vesborg P.C.K., Seger B. (2014). Iron-Treated NiO as a Highly Transparent p-Type Protection Layer for Efficient Si-Based Photoanodes. J. Phys. Chem. Lett..

[52] Dhull N., Kaur G., Gupta V., Tomar M. (2018). Development of Nanostructured Nickel Oxide Thin Film Matrix by Rf Sputtering Technique for the Realization of Efficient Bioelectrode. Vacuum.

[53] Reddy A.M., Reddy A.S., Lee K.-S., Reddy P.S. (2011). Growth and Characterization of NiO Thin Films Prepared by DC Reactive Magnetron Sputtering. Solid State Sci..

[54] Do H.H., Nguyen K.B., Nguyen P.N., Pham H.P. (2025). Facile One-Step Radio Frequency Magnetron Sputtering of Ni/NiO on Stainless Steel for an Efficient Electrode for Hydrogen Evolution Reaction. Beilstein J. Nanotechnol..

[55] Wang F., Jia J., Zhao W., Zhang L., Ma H., Li N., Chen Y. (2022). Preparation and Electrochromic Properties of NiO and ZnO-Doped NiO Thin Films. Mater. Sci. Semicond. Process..

[56] Oh S., Jun Y.-K., Kim N.-H. (2025). Magnetron-Sputtered and Rapid-Thermally Annealed NiO:Cu Thin Films on 3D Porous Substrates for Supercapacitor Electrodes. Energies.

[57] Zhang L., Chen Z., Ma H. (2024). Effect of Sputtering Pressure and Oxygen Content on the Electrochromic Properties of NiO Films by DC Magnetron Sputtering. Phys. B Condens. Matter.

[58] Lin C.-W., Chung W.-C., Zhang Z.-D., Hsu M.-C. (2017). P-Channel Transparent Thin-Film Transistor Using Physical-Vapor-Deposited NiO Layer. Jpn. J. Appl. Phys..

[59] Wang H., Wang Y., Wang X. (2012). Pulsed Laser Deposition of the Porous Nickel Oxide Thin Film at Room Temperature for High-Rate Pseudocapacitive Energy Storage. Electrochem. Commun..

[60] Farha A.H. (2023). Structural and Optical Characteristics of NiO Films Deposited Using the PLD Technique. Mater. Sci. Technol..

[61] Reddy G.K., Nagaraju P., Reddy G.L.N., Ghosal P., Reddy M.V.R. (2022). Growth and Characterization of Electron Beam Evaporated NiO Thin Films for Room Temperature Formaldehyde Sensing. Sens. Actuators A Phys..

[62] Hossain M.I., Aissa B. (2024). Electron-Beam-Evaporated Nickel Oxide Thin Films for Application as a Hole Transport Layer in Photovoltaics. Processes.

[63] Sahu D.R., Wu T.-J., Wang S.-C., Huang J.-L. (2017). Electrochromic Behavior of NiO Film Prepared by E-Beam Evaporation. J. Sci. Adv. Mater. Devices.

[64] Sahu D.R., Lee Y.-H., Wu T.-J., Wang S.-C., Huang J.-L. (2020). Synthesis and Electrochromic Property Improvement of NiO Films for Device Applications. Thin Solid Films.

[65] GangaReddy K., Reddy M.V.R. (2023). Physical Vapour Deposition of Zn^2+^ Doped NiO Nanostructured Thin Films for Enhanced Selective and Sensitive Ammonia Sensing. Mater. Sci. Semicond. Process..

[66] Benedet M., Maccato C., Pagot G., Invernizzi C., Sada C., Di Noto V., Rizzi G.A., Fois E., Tabacchi G., Barreca D. (2023). Growth of NiO Thin Films in the Presence of Water Vapor: Insights from Experiments and Theory. J. Phys. Chem. C.

[67] Han S.W., Kim I.H., Kim D.H., Park K.J., Park E.J., Jeong M.-G., Kim Y.D. (2016). Temperature Regulated-Chemical Vapor Deposition for Incorporating NiO Nanoparticles into Mesoporous Media. Appl. Surf. Sci..

[68] Sialvi M.Z., Mortimer R.J., Wilcox G.D., Teridi A.M., Varley T.S., Wijayantha K.G.U., Kirk C.A. (2013). Electrochromic and Colorimetric Properties of Nickel (II) Oxide Thin Films Prepared by Aerosol-Assisted Chemical Vapor Deposition. ACS Appl. Mater. Interfaces.

[69] Wang H., Wu G., Cai X.P., Zhao Y., Shi Z.F., Wang J., Xia X.C., Dong X., Zhang B.L., Ma Y. (2012). Effect of Growth Temperature on Structure and Optical Characters of NiO Films Fabricated by PA-MOCVD. Vacuum.

[70] Mostafa A.M., Mwafy E.A. (2020). The Effect of Laser Fluence for Enhancing the Antibacterial Activity of NiO Nanoparticles by Pulsed Laser Ablation in Liquid Media. Environ. Nanotechnol. Monit. Manag..

[71] Gondal M.A., Saleh T.A., Drmosh Q.A. (2012). Synthesis of Nickel Oxide Nanoparticles Using Pulsed Laser Ablation in Liquids and Their Optical Characterization. Appl. Surf. Sci..

[72] Hmmoodi A.M., Nayef U.M., Rasheed M. (2025). Synthesis of NiO Nanoparticles Using Laser Ablation in Liquid for Photodetector Application. Plasmonics.

[73] Safa M., Dorranian D., Masoudi A.A., Matin L.F. (2019). Characterizing Nickel Oxide Nanostructures Produced by Laser Ablation Method: Effects of Laser Fluence. Appl. Phys. A.

[74] Khashan K.S., Sulaiman G.M., Hamad A.H., Abdulameer F.A., Hadi A. (2017). Generation of NiO Nanoparticles via Pulsed Laser Ablation in Deionised Water and Their Antibacterial Activity. Appl. Phys. A.

[75] Khan S.Z., Yuan Y., Abdolvand A., Schmidt M., Crouse P., Li L., Liu Z., Sharp M., Watkins K.G. (2009). Generation and Characterization of NiO Nanoparticles by Continuous Wave Fiber Laser Ablation in Liquid. J. Nanopart. Res..

[76] Rahman A., Guisbiers G. (2024). Synthesis of Nickel-Based Nanoparticles by Pulsed Laser Ablation in Liquids: Correlations between Laser Beam Power, Size Distribution and Cavitation Bubble Lifetime. Metals.

[77] Alrebdi T.A., Ahmed H.A., Alsubhe E., Alkallas F.H., Mwafy E.A., Adel Pashameah R., Toghan A., Mostafa A.M. (2022). Synthesis of NiO-PVA Nanocomposite by Laser Assisted-Method and Its Characterization as a Novel Adsorbent for Removal Phosphate from Aqueous Water. Opt. Laser Technol..

[78] Joseph T., Thomas J., Thomas T., Thomas N. (2022). Electrocatalytic Activity Enhancement Using Graphene-Metal Oxide Nanocomposites for the Ultra Low Level Detection of Biomolecules. J. Electrochem. Soc..

[79] Zeng G., Li W., Ci S., Jia J., Wen Z. (2016). Highly Dispersed NiO Nanoparticles Decorating Graphene Nanosheets for Non-Enzymatic Glucose Sensor and Biofuel Cell. Sci. Rep..

[80] Song R., Zhang H., Lv J. (2023). NiO Nanoparticle-Decorated Graphene Oxide Nanosheets Modified Glassy Carbon Electrode for Sensitive Electrochemical Detection of Pethidine. Int. J. Electrochem. Sci..

[81] Kiran A., Hussain S., Ahmad I., Imran M., Saqib M., Parveen B., Munawar K.S., Mnif W., Huwayz M.A., Alwadai N. (2024). Green Synthesis of NiO and NiO@graphene Oxide Nanomaterials Using Elettaria Cardamomum Leaves: Structural and Electrochemical Studies. Heliyon.

[82] Ramu A.G., Umar A., Ibrahim A.A., Algadi H., Ibrahim Y.S.A., Wang Y., Hanafiah M.M., Shanmugam P., Choi D. (2021). Synthesis of Porous 2D Layered Nickel Oxide-Reduced Graphene Oxide (NiO-rGO) Hybrid Composite for the Efficient Electrochemical Detection of Epinephrine in Biological Fluid. Environ. Res..

[83] Krishnasamy V., Nair G.G., Sha M.S., Kannan K., Al-Maadeed S., Muthalif A.G.A., Sadasivuni K.K. (2023). A Promising Electrochemical Sensor Platform for the Detection of Dopamine Using CuO-NiO/rGO Composite. Macromol. Symp..

[84] Gao J., He P., Yang T., Zhou L., Wang X., Chen S., Lei H., Zhang H., Jia B., Liu J. (2019). Electrodeposited NiO/Graphene Oxide Nanocomposite: An Enhanced Voltammetric Sensing Platform for Highly Sensitive Detection of Uric Acid, Dopamine and Ascorbic Acid. J. Electroanal. Chem..

[85] Zhao L., Yu J., Yue S., Zhang L., Wang Z., Guo P., Liu Q. (2018). Nickel Oxide/Carbon Nanotube Nanocomposites Prepared by Atomic Layer Deposition for Electrochemical Sensing of Hydroquinone and Catechol. J. Electroanal. Chem..

[86] Wali R., Moulaee K., Qasymeh M., Maalej R., Neri G. (2024). Atomic Layer Deposition of NiO-Coated CNT Nanocomposites: Tailoring Electrochemical Properties for Salivary Lactate Detection. J. Electroanal. Chem..

[87] Wan Y., Zheng Y.F., Zhou B., Song X.C. (2018). An Innovative Electrochemical Sensor Ground on NiO Nanoparticles and Multi-Walled Carbon Nanotubes for Quantitative Determination of Nitrite. J. Nanosci. Nanotechnol..

[88] Liu Z., Shen Y. (2022). Synthesis of NiO-, CuO-, and Co_3_O_4_-Decorated Carbon Nanotubes for Electrochemical Detection and Conversion of Glucose. ACS Appl. Energy Mater..

[89] Ding H., Tao W. (2021). Synthesis of NiO-CNTs Nanocomposite for Modification of Glassy Carbon Electrode and Application for Electrochemical Determination of Fentanyl as an Opioid Analgesic Drug. Int. J. Electrochem. Sci..

[90] Zhang M., Wang Y., Qi Z., You B., Wang B., Wu Y., Wang Y., Zhang Z. (2025). Non-Invasive Salivary Glucose Sensing Technology and Performance Study Based on NiO/Multi-Walled Carbon Nanotube Composite Structures. Microchem. J..

[91] Ali A., Khalid A., Sajid A., Akyürekli S., Ahmed A.Y., Bayach I., Buzid A., Alharthi A.F. (2025). NiO@CNTs Wrapped Graphene Oxide Nanohybrid towards Simultaneous Electrochemical Detection of Ascorbic Acid and Uric Acid. Inorg. Chem. Commun..

[92] Narayanan K., Murugan K.S., Natarajan T.S. (2025). Ag@NiO/g-C_3_N_4_ Nanocomposite: An Efficient and High-Performance Electrochemical Sensor for Acetaminophen Detection. New J. Chem..

[93] Ullah M., Kanjariya P., Rekha M.M., Kundlas M., Prasad G.V.S., Chahar M., Algahtani A., Tirth V., Zhengxin L. (2025). Fabrication of an Electrochemical Sensor Based on G-C_3_N_4_–NiO Nanocomposite for Sensitive and Selective Detection of Hydrogen Peroxide. J. Mater. Sci. Mater. Electron..

[94] Ngo Y.-L.T., Chung J.S., Hur S.H. (2020). Multi-Functional NiO/g-C_3_N_4_ Hybrid Nanostructures for Energy Storage and Sensor Applications. Korean J. Chem. Eng..

[95] Selvarajan S., Suganthi A., Rajarajan M. (2018). Fabrication of G-C_3_N_4_/NiO Heterostructured Nanocomposite Modified Glassy Carbon Electrode for Quercetin Biosensor. Ultrason. Sonochem..

[96] Gong W., Zou J., Zhang S., Zhou X., Jiang J. (2016). Nickel Oxide and Nickel Co-Doped Graphitic Carbon Nitride Nanocomposites and Its Octylphenol Sensing Application. Electroanalysis.

[97] Li X., Wang Y., Tian W., Cao J. (2019). Graphitic Carbon Nitride Nanosheets Decorated Flower-like NiO Composites for High-Performance Triethylamine Detection. ACS Omega.

[98] Chen X., Yang Z., Ai L., Zhou S., Fan H., Ai S. (2022). Signal-off Photoelectrochemical Aptasensor for *S. aureus* Detection Based on Graphite-like Carbon Nitride Decorated with Nickel Oxide. Electroanalysis.

[99] Lotfi Z., Gholivand M.B., Shamsipur M. (2021). Non-Enzymatic Glucose Sensor Based on a g-C_3_N_4_/NiO/CuO Nanocomposite. Anal. Biochem..

[100] Shu Y., Yan Y., Chen J., Xu Q., Pang H., Hu X. Ni and NiO Nanoparticles Decorated Metal–Organic Framework Nanosheets: Facile Synthesis and High-Performance Nonenzymatic Glucose Detection in Human Serum. https://pubs.acs.org/doi/abs/10.1021/acsami.7b07501.

[101] Wang L., Xie Y., Wei C., Lu X., Li X., Song Y. (2015). Hierarchical NiO Superstructures/Foam Ni Electrode Derived from Ni Metal-Organic Framework Flakes on Foam Ni for Glucose Sensing. Electrochim. Acta.

[102] Jia S., Wang Q., Wang S. (2021). Ni-MOF/PANI-Derived CN-Doped NiO Nanocomposites for High Sensitive Nonenzymic Electrochemical Detection. J. Inorg. Organomet. Polym..

[103] Ambrogi E.K., Li Y., Chandra P., Mirica K.A. (2025). Employing Triphenylene-Based, Layered, Conductive Metal–Organic Framework Materials as Electrochemical Sensors for Nitric Oxide in Aqueous Media. ACS Sens..

[104] Wang J., Zhao J., Yang J., Cheng J., Tan Y., Feng H., Li Y. (2020). An Electrochemical Sensor Based on MOF-Derived NiO@ZnO Hollow Microspheres for Isoniazid Determination. Microchim. Acta.

[105] Gao F., Tu X., Ma X., Xie Y., Zou J., Huang X., Qu F., Yu Y., Lu L. (2020). NiO@Ni-MOF Nanoarrays Modified Ti Mesh as Ultrasensitive Electrochemical Sensing Platform for Luteolin Detection. Talanta.

[106] Alizadeh Z., Mazloum-Ardakani M., Asadpour F., Yavari M. (2023). Highly Efficient Enzyme-Free Glutamate Sensors Using Porous Network Metal–Organic Framework-Ni-NiO-Ni-Carbon Nanocomposites. ACS Appl. Mater. Interfaces.

[107] Iftikhar T., Aziz A., Ashraf G., Xu Y., Li G., Zhang T., Asif M., Xiao F., Liu H. (2022). Engineering MOFs Derived Metal Oxide Nanohybrids: Towards Electrochemical Sensing of Catechol in Tea Samples. Food Chem..

[108] Sun Q., Zhang Y., Gao P., Pu H., Yin M., Liang X., Yin W., Fa H. (2023). Three-Dimensional NiO/Co_3_O_4_@C Composite for High-Performance Non-Enzymatic Glucose Sensor. Anal. Sci..

[109] Feng L., Zou M., Lv X., Min X., Lin X., Ni Y. (2022). Facile Synthesis of ZIF-67C@RGO/NiNPs Nanocomposite for Electrochemical Non-Enzymatic Sensing Platform of Nitrite. Microchem. J..

[110] Zhang Z., Wang J., Guo S., Wang X. (2025). C-ZIF-8 Modified NiO Photocathode and Enhanced Photosensitizer Signal Amplification for Ultra-Sensitive Photoelectrochemical Detection of Lead Ions. Anal. Methods.

[111] Yao W., Guo H., Liu H., Li Q., Wu N., Li L., Wang M., Fan T., Yang W. (2020). Highly Electrochemical Performance of Ni-ZIF-8/ N S-CNTs/CS Composite for Simultaneous Determination of Dopamine, Uric Acid and L-Tryptophan. Microchem. J..

[112] Zhang W., Zong L., Liu S., Pei S., Zhang Y., Ding X., Jiang B., Zhang Y. (2019). An Electrochemical Sensor Based on Electro-Polymerization of Caffeic Acid and Zn/Ni-ZIF-8–800 on Glassy Carbon Electrode for the Sensitive Detection of Acetaminophen. Biosens. Bioelectron..

[113] Xie A., Yuan B., Lin J., Pan J., Li M., Wang J., Jiang S., Zhu S., Luo S. (2023). Highly Sensitive and Selective Electrochemical Sensor Based on ZIF-67-Derived Co_3_O_4_-LaMn_0.5_Ni_0.5_O_3_/MWCNTs for Simultaneous Detection of Protocatechuic Acid and Ascorbic Acid. Surf. Interfaces.

[114] Liang H., Luo Y., Xiao Y., Chen R., Wang L., Song Y. (2024). Ni/NiO/Carbon Derived from Covalent Organic Frameworks for Enzymatic-Free Electrochemical Glucose Sensor. Ceram. Int..

[115] Hilal M., Ali Y., Cai Z., Kim H., Abdo H.S., Alnaser I.A., Hwang Y. (2025). Synergistic MXene@NiO-ZnO Heterostructures via Dual-Pressure Hydrothermal Synthesis for High-Performance Photoelectrochemical Glucose Sensing. Ceram. Int..

[116] Saraswathi K.A., Reddy M.S.B., Jayarambabu N., Harish C.M., Venkateswara Rao K., Venkatappa Rao T. (2025). NiO Embedded PANI /Ti_3_C_2_T_*x*_ MXene Detector for Electrochemical Enzyme Free Glucose Detection. Surf. Interfaces.

[117] Yelmai S.W., Alom N., Han T.K., Saidur R., Jamal M. (2023). Fabrication of High-Performance pH Sensor Based on NiO/Mxene/PANI Modified Sensing Platform. Meet. Abstr..

[118] Siva Ranjani R., Wilson J., Madhu Malar M., Faiyazuddin M., Gowri S. (2025). Green Synthesized ZnO/NiO Nanocomposites Decorated Tragacanth Gum for Electrochemical Detection of Riboflavin. Microchem. J..

[119] Hyder A., Ali A., Buledi J.A., Memon R., Al-Anzi B.S., Memon A.A., Kazi M., Solangi A.R., Yang J., Thebo K.H. (2024). A NiO-Nanostructure-Based Electrochemical Sensor Functionalized with Supramolecular Structures for the Ultra-Sensitive Detection of the Endocrine Disruptor Bisphenol S in an Aquatic Environment. Phys. Chem. Chem. Phys..

[120] Nagarajan A., Sethuraman V., Sridhar T.M., Sasikumar R. (2023). Development of Au@NiO Decorated Polypyrrole Composite for Non-Enzymatic Electrochemical Sensing of Cholesterol. J. Ind. Eng. Chem..

[121] Chufamo Jikamo S., Haile Habtemariam T., Heliso Dolla T. (2023). Polyaniline-ZnO−NiO Nanocomposite Based Non-Enzymatic Electrochemical Sensor for Malathion Detection. Electroanalysis.

[122] Chowdhury M.S.H., Rahman Khan M.M., Deb B., Shohag M.R.H., Rahman S., Rahman M.d.M., Alzahrani K.A., Rahman M.M., Bhuyan M.M., Jeong J.-H. (2025). Fabrication of Poly (Ani-Co-Py)/NiO Composites with Superb Photocatalytic Performance and Effective p-Nitrophenol Sensor. J. Inorg. Organomet. Polym..

[123] Thenrajan T., Nagappan S., Kundu S., Wilson J. (2023). Nickel Iron Based Layered Double Hydroxides as Effective Electrochemical Sensor towards Epicatechin. Inorg. Chem. Commun..

[124] Beitollahi H., Dourandish Z., Tajik S., Sharifi F., Jahani P.M. (2022). Electrochemical Sensor Based on Ni-Co Layered Double Hydroxide Hollow Nanostructures for Ultrasensitive Detection of Sumatriptan and Naproxen. Biosensors.

[125] Hai B., Zou Y. (2015). Carbon Cloth Supported NiAl-Layered Double Hydroxides for Flexible Application and Highly Sensitive Electrochemical Sensors. Sens. Actuators B Chem..

[126] Kokulnathan T., Wang T.-J., Ashok Kumar E., Ahmed F. (2022). Construction of Nickel Cobalt-Layered Double Hydroxide/Functionalized–Halloysite Nanotubes Composite for Electrochemical Detection of Organophosphate Insecticide. Chem. Eng. J..

[127] Wu Y.-T., Tsao P.-K., Chen K.-J., Lin Y.-C., Aulia S., Chang L.-Y., Ho K.-C., Chang C., Mizuguchi H., Yeh M.-H. (2021). Designing Bimetallic Ni-Based Layered Double Hydroxides for Enzyme-Free Electrochemical Lactate Biosensors. Sens. Actuators B Chem..

[128] Yang J., Xu L. (2025). NiCu-Layered Double Hydroxide–Modified CuO Nanorods for Enhanced Non-Enzymatic Glucose Sensing. Microchim. Acta.

[129] Kokulnathan T., Wang T.-J., Ahmed F., Arshi N. (2023). Fabrication of Flower-like Nickel Cobalt-Layered Double Hydroxide for Electrochemical Detection of Carbendazim. Surf. Interfaces.

